# Longitudinal characterization of primary osteosarcoma and derived subcutaneous and orthotopic relapsed patient-derived xenograft models

**DOI:** 10.3389/fonc.2023.1166063

**Published:** 2023-06-12

**Authors:** Maria Eugenia Marques da Costa, Robin Droit, Pierre Khneisser, Anne Gomez-Brouchet, Tiphaine Adam-de-Beaumais, Marie Nolla, Nicolas Signolles, Jacob Torrejon, Bérangère Lombard, Damarys Loew, Olivier Ayrault, Jean-Yves Scoazec, Birgit Geoerger, Gilles Vassal, Antonin Marchais, Nathalie Gaspar

**Affiliations:** ^1^ INSERM U1015, Université Paris-Saclay, Villejuif, France; ^2^ Department of Pediatric and Adolescent Oncology, Gustave Roussy Cancer Campus, Université Paris-Saclay, Villejuif, France; ^3^ Department of Medical Biology and Pathology, Gustave Roussy Cancer Campus, Villejuif, France; ^4^ Department of Pathology, IUCT-Oncopole, CHU Toulouse and University Toulouse, Pharmacology and Structural Biology Institute, CNRS UMR5089, Toulouse, France; ^5^ Department of Pediatric Hemato-oncology, CHU Toulouse, Toulouse, France; ^6^ Institut Curie, PSL Research University, CNRS UMR, INSERM, Orsay, France; ^7^ Université Paris Sud, Université Paris-Saclay, CNRS UMR, INSERM, Orsay, France; ^8^ Institut Curie, PSL Research University, Centre de Recherche, Laboratoire de Spectrométrie de Masse Protéomique, Paris, France

**Keywords:** osteosarcoma, patient-derived xenograft, bone, omics, RNAseq

## Abstract

Osteosarcoma is a rare bone cancer in adolescents and young adults with a dismal prognosis because of metastatic disease and chemoresistance. Despite multiple clinical trials, no improvement in outcome has occurred in decades. There is an urgent need to better understand resistant and metastatic disease and to generate *in vivo* models from relapsed tumors. We developed eight new patient-derived xenograft (PDX) subcutaneous and orthotopic/paratibial models derived from patients with recurrent osteosarcoma and compared the genetic and transcriptomic landscapes of the disease progression at diagnosis and relapse with the matching PDX. Whole exome sequencing showed that driver and copy-number alterations are conserved from diagnosis to relapse, with the emergence of somatic alterations of genes mostly involved in DNA repair, cell cycle checkpoints, and chromosome organization. All PDX patients conserve most of the genetic alterations identified at relapse. At the transcriptomic level, tumor cells maintain their ossification, chondrocytic, and trans-differentiation programs during progression and implantation in PDX models, as identified at the radiological and histological levels. A more complex phenotype, like the interaction with immune cells and osteoclasts or cancer testis antigen expression, seemed conserved and was hardly identifiable by histology. Despite NSG mouse immunodeficiency, four of the PDX models partially reconstructed the vascular and immune-microenvironment observed in patients, among which the macrophagic TREM2/TYROBP axis expression, recently linked to immunosuppression. Our multimodal analysis of osteosarcoma progression and PDX models is a valuable resource to understand resistance and metastatic spread mechanisms, as well as for the exploration of novel therapeutic strategies for advanced osteosarcoma.

## Introduction

Osteosarcoma is a rare bone cancer in adolescents and young adults with a dismal prognosis linked to metastatic disease (at diagnosis or relapse) and chemoresistance (poor histological response to first-line neoadjuvant chemotherapy) (1–3). Despite multiple phase III trials at diagnosis and phase II trials in relapsed/refractory osteosarcomas, no outcome improvement has occurred in decades (4). Most trials targeting specific pathways lacked patient selection based on molecular evidence (1–4). No molecular stratification or biomarkers have reached the clinical stage as prognostic factors or guided the development of new targeted therapies. Osteosarcoma’s heterogeneity probably explains the difficulty in defining shared molecular signatures in the high-risk patient population. In the future, a better understanding of tumor genetic instability (5) and microenvironment complexity (6, 7) should facilitate the selection of relevant markers. Recent studies, based on omics analyses, have highlighted interesting molecular pathways involving cancer testis antigens, *PPARG*, *HDAC4*, or *TIGIT* (8–11). In this context, well-characterized preclinical models, representative of osteosarcoma complexity, heterogeneity, chemo-resistant, and metastatic behaviors, should rationalize and accelerate pre-clinical testing of innovative, effective therapies and the identification of companion biomarkers (12). In the last few years, patient-derived xenograft (PDX) models have been developed to better mimic the biology and heterogeneity of human tumors (13). PDX models have been shown to closely recapitulate the genomic alterations present in the tumor of origin (14). However, few subcutaneous and orthotopic PDX models have yet been described for osteosarcoma, either from diagnosis or relapse samples, partly due to the low engraftment rate and the relatively long time required for tumor establishment in these models (12, 14–16).

Here, we present the longitudinal and multimodal characterization of primary tumors and derived subcutaneous and orthotopic paratibial osteosarcoma PDX models developed in NOD.Cg-Prkdc^scid^IL2rg^tm1Wjl^/SzJ (NSG) mice at Gustave Roussy Cancer Campus (Villejuif, France), from refractory/relapsed osteosarcoma samples of adolescents and young adults included in the MAPPYACTS trial (Molecular Profiling for Pediatric and Young Adult Cancer Treatment Stratification) (17).

## Results

**Figure 1:**
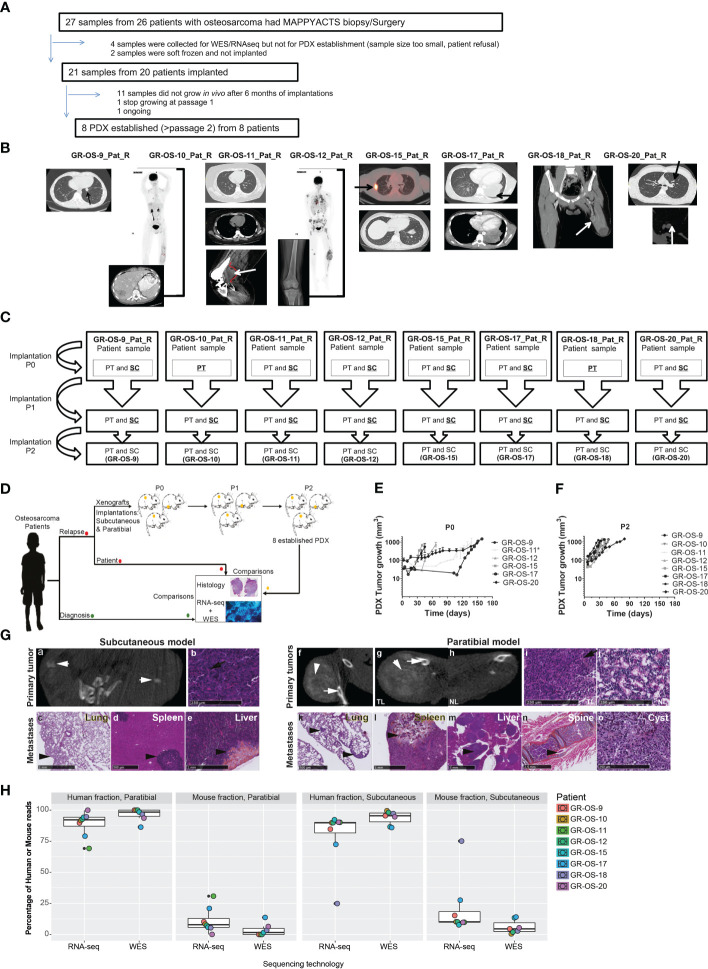
Tumor engraftment, radiological, and morphological characteristics of the eight osteosarcoma PDX models implanted subcutaneously and paratibially in NSG mice. **(A)** Origin of osteosarcoma patient samples used for PDX development. **(B)** Patient biopsy/surgery origin site used for PDX development. **(C)** PDX development from patient sample implanted at P0 until implantation at P2. The underlined implantation method represents the method used in each passage to perform the next passage. At each passage, the method underlined was used to perform the next passage. **(D)** Experimental design of PDX development; **(E, F)** Tumor growth of subcutaneous models in passage 0 and passage 2, respectively. *PDX was developed from a patient-frozen sample. **(G)** Radiological and morphological characteristics of the PDX GR-OS-15 primary tumor and metastases: CT scan images from the subcutaneous tumor (a) and the leg bearing the paratibial tumor (f and g) and normal leg (h), obtained with an Ivis Spectrum scan. Histology was performed for the primary tumor of the subcutaneous model (b), the paratibial tumor (i) and the normal leg (j), and the metastases of the subcutaneous (c–e) and paratibial (k–o) models. The short white arrows show osteocondensation, the long white arrows osteolysis, the long black arrows osteoid matrix (orange color), and the short black arrows mark metastases. TL, tumor leg; NL, normal leg. **(H)** Boxplot of the percentage of reads aligned on tumor cells (human fraction) or the microenvironment (mouse fraction) in PDX inferred either from the Xenome RNA-Seq or WES analyses.

Eight subcutaneous and eight paratibial osteosarcoma PDX models were established from eight adolescent and young adult cases of refractory/relapsed osteosarcomas and characterized at multiple levels. They were compared to their matched patient tumors sampled at initial diagnosis and relapse (**Figures 1A–C**; **Supplementary Table SI**).

Eight osteosarcoma samples had tumor engraftment in both settings at P0 and were considered established when they reached at least passage 2 (P2), leading to a PDX-establishment rate of 8/20 (one more model is ongoing at P2) for tumor samples implanted subcutaneously and paratibially. The tumor take rate was better for subcutaneous osteosarcoma PDX models (sc-PDX), with 100% for all models, than for paratibial models (pt-PDX), which ranged from 56% to 100% (median 60%), depending on the PDX model (**Supplementary Table SII**). The first subcutaneous model (GR-OS-9) was also implanted in Swiss nude mice, with a slower tumor development than in NSG mice (**Supplementary Figure S1**). This mouse strain was thus not further employed.

### Osteosarcoma PDX local and metastatic behavior mimic human tumors behavior at local and metastatic level

Subcutaneous (sc-) and paratibial (pt-) PDXs harbored different tumor behaviors due to their distinct implantation sites (**Figure 1D**). The bone microenvironment seems to be reproduced in PDX at both implantation sites, but pt-PDX was better at mimicking human primary tumor behavior than sc-PDX models. For sc-PDX, although the time between implantation and tumor growth detection varied by tumor sample in early passages (shorter for GR-OS-18), similar behaviors were seen after passage P2 (**Figures 1E, F**). sc-PDX growth could be detected from days 7 to 18 (median 13). The time to reach a tumor volume around 900 mm^3^ was 26–62 days (median 41) after implantation (**Supplementary Table SII**). The median sc-PDX growth doubling time was 5 days (range 4–7) (**Figure 1F**). Detection of calcification on a computer tomography (CT) scan was observed in sc-PDX, although to a lesser extent compared to the corresponding pt-PDX (**Figures 1G-a, f, g, h**; **Supplementary Figure S2**). Only three sc-PDX developed metastases, within the lungs (GR-OS-12, GR-OS-15) (**Figure 1G–c**), but also at unusual sites, the spleen (GR-OS-15, GR-OS-20), and the liver (GR-OS-15) (**Figures 1G-d, e**; **Table 1**).

**Table 1:** Histological characteristics of the eight subcutaneous and paratibial osteosarcoma PDX models.

PDX ID	Implantation site	First tumor detection median time (days)	Median time Detection- Sacrifice (days)	TD (doubling time - days)		% Tumor take	Tumor aspect	Growth localization	PDX Cells culture
				P0	P2				
**GR-OS-9**	Subcutaneous	24 (11-67)	28 (10-48)	5.2	4	100	Calcified (++)	in the two flanks	Growth stopped P2 +++ cells adherence
	Paratibial	27 (11-42)	26 (13-28)	–	–	56	Calcified (+++)	Tibia, Femur and pelvis (close to the bone)	Growth stopped P1 +++ cells adherence
**GR-OS-10**	Subcutaneous	25 (19-26)	35 (22-52)	–	5.5	100	Calcified (++)	in the two flanks	Growth after P2 ++ cells adherence
	Paratibial	69 (40-97)	58 (34-80)	–	–	100	Calcified (++++) and viscous	Tibia (Inside the bone)	Growth after P2 ++ cells adherence
**GR-OS-11**	Subcutaneous	25 (24-109)	29 (21-42)	14	7	100	Calcified (+)	Tibia (Inside the bone)	Growth after P2 + cells adherence
	Paratibial	25 (19-42)	27 (21-62)	–	–	60	Calcified (++)	Tibia (Inside the bone)	Growth after P2 + cells adherence
**GR-OS-12**	Subcutaneous	14 (13-52)	19 (14-29)	8	5	100	Calcified (++)	in the two flanks	Growth after P2 ++ cells adherence
	Paratibial	33 (11-42)	20 (17-22)	–	–	60	Soft exterior and calcified (+++)	Tibia and femur (Inside the bone )	Growth after P2 ++ cells adherence
**GR-OS-15**	Subcutaneous	15 (10-27)	15.5 (7-28)	5	4.2	100	Calcified (+++)	in the two flanks	Growth after P2 ++ cells adherence
	Paratibial	27 (19-44)	28 (18-41)	–	–	83	Calcified (+++++)	Tibia (Inside the bone)	Growth after P2 ++ cells adherence
**GR-OS-17**	Subcutaneous	42 (35-70)	94 (12-125)	9	6.5	100	No Calcified	in the two flanks	Slow Growth (P0)
	Paratibial	74,5 (70-93)	71 (12-95)	–	–	80	Calcified (+)	Tibia (Inside the bone)	Slow Growth (P0)
**GR-OS-18**	Subcutaneous	19,5 (18-21)	9 (8-10)	–	5	100	Calcified (++)	in the two flanks	Growth after P2 + cells adherence
	Paratibial	19 (19-22)	32 (20-48)	–	–	100	Calcified (++)	Tibia and Femur (Inside the bone)	Growth after P2 + cells adherence
**GR-OS-20**	Subcutaneous	93,5 (27-156)	23 (17-63)	22	11	100	Calcified (+)	in the two flanks	–
	Paratibial	135 (53-217)	52 (43-60)	–	–	50	Calcified (+)	Tibia (Inside the bone)	–

HG, high grade; OB, osteoblastic; FB, fibroblastic; CB, chondroblastic; PS, pleomorphic sarcoma; NA, not applicable; ND, not done, (**−**), not present; (**+**), present.

In pt-PDX, tumor growth was more difficult to detect and follow due to tumor depth, and was revealed by clinically measurable swelling and bone alterations on CT scan, as is often observed in patients. pt-PDXs developed within the tibia with aggressive bone lesions, associated with both osteocondensation (aberrant new bone formation extending within the extra-osseous mass) and osteolysis (bone destruction), again as is observed in patients (**Figures 1G-f, g, h**; **Table 1**, **Supplementary Figure S2**). The degree of calcification varied between models from low (GR-OS-17) to high (GR-OS-15) and was confirmed histologically (**Supplementary Figure S2**; **Table 1**). One model (GR-OS-15) had macro-metastases detected by CT scan (**Supplementary Figure S2**); in all other cases (**Supplementary Figure S2**), metastases were detectable only by histology at the time of sacrifice in sites classically observed in patients, such as the lungs (GR-OS-12, GR-OS-15) and bones (GR-OS-10), or less frequently the liver (GR-OS-15) (**Figures 1G-k, l**; **Table 1**). Unusual spleen metastases were detected in all but two pt-PDX (GR-OS-11, GR-OS-20), which were apparently metastases-free at any site. The GR-OS-15_pt model also developed metastases in other unusual sites (e.g., ovary, spine) (**Figures 1G–m, n, o**; **Table 1**).

HES (hematoxilin–eosin–safranin) staining confirmed the high-grade osteosarcoma histology in all sc-PDX and pt-PDX models as observed in patients’ tumors (**Figure 1G**; **Table 1**). Morphology was predominantly osteoblastic with pleomorphic tumor cells and some degree of necrosis, including for the PDX originating from non-conventional telangiectatic osteosarcoma (GR-OS-10). A fibroblastic component was observed in the PDX issued from fibroblastic osteosarcoma (GR-OS-11), associated with a chondroblastic component only in the paratibial model. Osteoid matrix was observed in all PDX independent of the implantation site and associated with chondroblastic matrix for PDX models issued from osteosarcomatosis GR-OS-12 (**Figure 1G**; **Table 1**; **Supplementary Figure S2**; **Supplementary Table 1**). The median percentage of tumor cells in PDX inferred from RNA and whole exome (WES) sequencing was 84% ( ± 3%) (**Figure 1H**).

Sensitivity/resistance to chemotherapeutic agents and multi-kinase inhibitors used in osteosarcoma patients were tested *in vitro* in secondary PDX-derived cell cultures issued from 6/8 PDX at second passage (GR-OS-17 did not grow *in vitro* and GR-OS-20 was not performed) to allow comparison with known, well-established osteosarcoma cell lines (**Table 2**). All PDX-derived cell models showed slow cell growth and high adherence, leading to resistance to trypsinization. Drug testing confirmed higher 50% inhibiting concentrations (IC50s) for PDX-derived cells to chemotherapeutic agents commonly used in osteosarcoma (methotrexate, doxorubicin, cisplatin, mafosfamide, and etoposide) as compared to usual osteosarcoma cell lines. Resistance to methotrexate was observed in 5/6 PDX-derived cell models (IC50 >100 µM) at a much higher level than cell lines rendered resistant through continuous MTX exposure *in vitro* (IC50 2–4 µM) (18). Doxorubicin IC50 ranged from 0.17 to 1.12 µM, except for GR-OS-15, where the IC50 was in the nM range usually observed for osteosarcoma cell lines. *In vitro* sensitivity to multi-kinase inhibitors (cabozantinib, regorafenib, and pazopanib) differed between drugs in each model, with cabozantinib having the lowest IC50 and pazopanib the highest. Indeed, pazopanib IC50s were higher than 30 µM in 5/6 models, except for the GR-OS-12 model (IC50 = 6 µM) originating from a patient who had experienced a radiological partial response to this drug at relapse.

**Table 2:** IC50s of cell cultures derived from PDX compared to established cell lines parental and resistant to chemotherapy by MTS assay.

Cell models		IC50 (μM)							
		MIX	DOXO	ETOP	CISP	MAF	CABO	REGO	PAZO
**GR-OS-9**	from PDX		MRX7	o9Rf	XTRT	79Rf	ToR9	T7R7	> 100
**GR-OS-10**	from PDX		MRf	TfR7	TR99	95RT	fRM7	XXRT	> 100
**GR-OS-11**	from PDX	> 100	MRX9	9XRT	T2R7	XXRT	TXRm	ToR9	> 100
**GR-OS-12**	from PDX	> 100	MRTf	TRmo	7R9	fRMT	7RM7	TMRm	5R72
**GR-OS-15**	from PDX	> 100	MRM2	MRoX	9RX	MRm9	9RT	Tm	> 100
**GR-OS-18**	from PDX	MRMT	TRXT	5R9m	XR27	T5R9	7Rm7	TXR9	9MR5
**HOS**	Parental	MRM2	MRM7	MRf	2Ro	TXRf	5R7	ND	ND
	R/MTX	5	MRMf	MR5f	7R9o	T5Ro	7Rf5	ND	ND
	R/DOXO	MR2o	TTRX	ToM	9	T2R9	T9R5	ND	ND
**HOS-143B**	Parental	MRM2	MRM2	MR5o	TR5o	T2R9	T2R5	ND	ND
	R/MTX	2RT9	MRT	MRm	9Rm5	TXRf	X9	ND	ND
**Saos-2**	Parental	MRM7	MRM7	XRmf	2RXo	TfR5	To	ND	ND
	R/MTX	XRM7	MRT	7RXT	5R22	X9Rm	X9Rm	ND	ND
**Saos-2-B**	Parental	MRM7	MRM5	XRo	7RX	XMR9	fR7o	ND	ND
	R/MTX	TRm	MRM5	9Rf5	7Rm5	XT	T7R9	ND	ND
**MG-63**	Parental	MRM7	MRT	X	XR2o	T9R9	oR7	ND	ND
	R/MTX	XRo2	MRX2	mRoT	9R9o	X5RX	5	ND	ND
**U2OS**	Parental	MRM7	MRT	2R2	TM	99	5R5	ND	ND
**IOR/OS18**	Parental	TR9	MRTo	7Ro5	2R5X	XfRT9	5	ND	ND

Treatment was performed using methotrexate (MTX), doxorubicin (DOXO), etoposide (ETOP), cisplatin (CISP), mafosfamide (MAF), cabozantinib (CABO), regorafenib (REGO), and pazopanib (PAZO). In vitro tests were not performed for the PDX GR-OS-17 and GR-OS-20. A panel of colors was used to classify the models according to the IC50. Green IC50 <1 µM; yellow IC50 1–10 µM; orange IC50 10–100 µM; red IC50 >100 µM.

### Osteosarcoma PDX models recapitulate the genomic landscape of the human tumor

**Figure 2:**
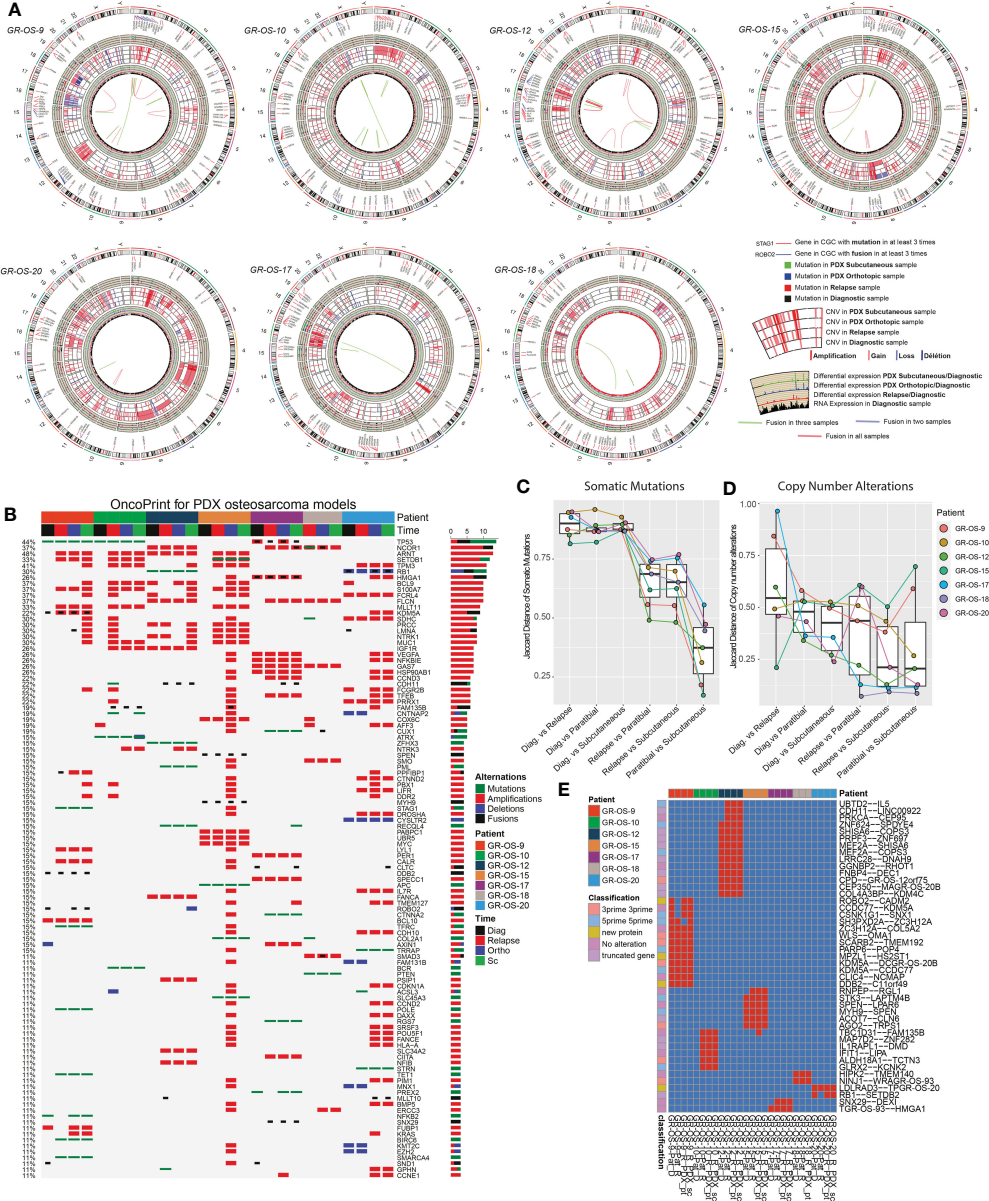
Genomic landscape of osteosarcoma subcutaneous and paratibial (orthotopic) PDX models compared to their derived relapsed metastatic tumor and the corresponding primary tumor at diagnosis. **(A)** Circos plot regrouping all the genetic alterations (somatic mutations, CNA, fusion transcripts, and expression levels). **(B)** Oncoprint of all the genetic alterations (somatic mutations, CNAs, and fusions) for the genes of the Cancer Gene Census (CGC). **(C)** Jaccard distance between times of samples for the somatic mutations. **(D)** Jaccard distance between times of samples for the CNA. **(E)** Conservation of the fusions through time and patients. Dia, diagnosis; Rel, relapse; pt, paratibial; sc, subcutaneous.

Primary sites of all eight pt- and sc-PDX samples and all matched human relapse samples were analyzed by WES and at the transcriptomic level to identify mutations, copy number alterations (CNA), and gene fusions and to quantify gene expression (**Figure 2A**). We applied the same analysis to the initial primary tumor biopsy at diagnosis of matched patients (except for GR-OS-11_Pat_D and GR-OS-18_Pat_D, where no patient DNA sample at diagnosis was available). For PDX samples, the genomes/transcriptomes were analyzed separately for the human (osteosarcoma clones) and mouse (tumor microenvironment) components using Xenome (except for paratibial GR-OS-11, with no patient DNA sample available).

At the genetic level, the main somatic driver mutations or deletions are all conserved in diagnosis, relapse and PDX models (**Figure 2B**). *TP53* mutations are observed consistently in GR-OS-9 and GR-OS-10 samples; *RB1* and *ZFHX3* mutations in GR-OS-12; a *RB1* homozygous deletion in GR-OS-20 (associated with a fusion *RB1::SETDB2*); and an *APC* heterozygotic mutation associated with the *MUTYH* germline mutation in GR-OS-15 (**Figure 2B**). Either at relapse or after xenografting, we observed on average 1.48 ( ± 0.27) times more new mutations. The genetic profiles of paratibial and subcutaneous PDX models for all models were more similar to the matched patient tumor at relapse than to those at diagnosis (**Figure 2C**). Matched subcutaneous and orthotopic models shared highly similar genetic backgrounds despite transplants originating from distinct tumor mouse fragments and experiencing several passages in different mice. Interestingly, for several patients, we observed the emergence of clones carrying new somatic heterozygous mutations at relapse that were conserved in PDX: *BIRC6* (GR-OS-9, NM_016252:p.L4413F), *POLE* (GR-OS-9, NM_006231:p.D23Y), *STAG1* (GR-OS-9, NM_005862:p.N784I), *TFRC* (GR-OS-9, NM_001313966: p.R57S), *SMARCA4* (GR-OS-9, NM_001128845:p.M949I) *TET1* (GR-OS-9, NM_030625:p.R1783Q), *BCR* (GR-OS-10, NM_004327: p.T1018A), *RECQL4* (GR-OS-12, NM_004260:p.R482C), *PML* (GR-OS-12, NM_002675:p.D412N), *SETDB1* (GR-OS-15, NM_001145415:p.V274D), *SLC45A3* (GR-OS-15, NM_033102:p.G231R), *RGS7* (GR-OS-17, NM_001282773:p.R308S), *STRN* (GR-OS-20, NM_003162:p.K516N), and *TRRAP* (GR-OS-20, NM_001244580:p.E36X). Most of these genes are involved in cell cycle checkpoints, chromosome organization, or DNA damage repair, which might provide clues about the mechanism of tumor cell evasion from tumor suppressors, therapy, or the patient’s immune response. Inspection of the allele frequencies of somatic mutations during disease progression from primary to relapsed tumor and in the pure tumoral fraction of PDX models (post-Xenome filtering) suggests that in two patients (GR-OS-10 and GR-OS-17), metastases arise from polyclonal populations with clonal branching pre-diagnosis (data not shown).

As a corollary of the putative greater instability in metastases due to the new mutations described above, we observed a greater accumulation of CNA between diagnosis and relapse than between relapse and PDX at the genomic level (**Figure 2D**; **Supplementary Figure S3C**), with major new gain and amplification events post-diagnosis (**Supplementary Figure S3B**). However, we observed that PDX models conserved the structural alterations inherited from the relapsed tumor cells with few additional alterations in cancer genes (**Figure 2D**; **Supplementary Figures S3A, B**). Several models showed conserved focal amplifications of *MYC* (GR-OS-15), *IGF1R* (GR-OS-10, GR-OS-12), *VEGFA* (GR-OS-17), or gain of the 4p12 amplicon with *VEGFR, PDGFR* associated with a gain of *CCND3* (chromosome 6p21.1) (GR-OS-11). Interestingly, in almost every model, we identified focal amplifications of either *NCOR1* (involved in the histone deacethylase complex in GR-OS-12, GR-OS-17, and GR-OS-18), or *SETDB1* (a histone methyltransferase that specifically trimethylates ‘Lys-9’ of histone H3 in GR-OS-9, GR-OS-10, and GR-OS-15), two genes mediating repressive histone modifications through interaction with HDAC proteins.

Despite massive structural genomic alterations, no recurrent fusions were described in osteosarcoma tumors, but fusions have been rarely studied through disease progression or after grafting in mice. To predict fusions, we used a combination of four prediction tools and filtered out fusions detected with less than three tools. The selected fusions were then searched again in all the samples with the FusionInspector tool to increase the detection sensitivity. Based on this strategy, we did not detect recurrent fusions within the models. However, we detected in some models fusions conserved from diagnosis to PDX (n = 27) or from relapse to PDX (n = 13) (**Figure 2E**). The PDX model GR-OS-17 presents a typical TP53 fusion with a breakpoint in the first exon, as reported by Dupain et al. (19).

### Tumor cells in PDX models express similar transcriptomic programs than patient tumors

**Figure 3:**
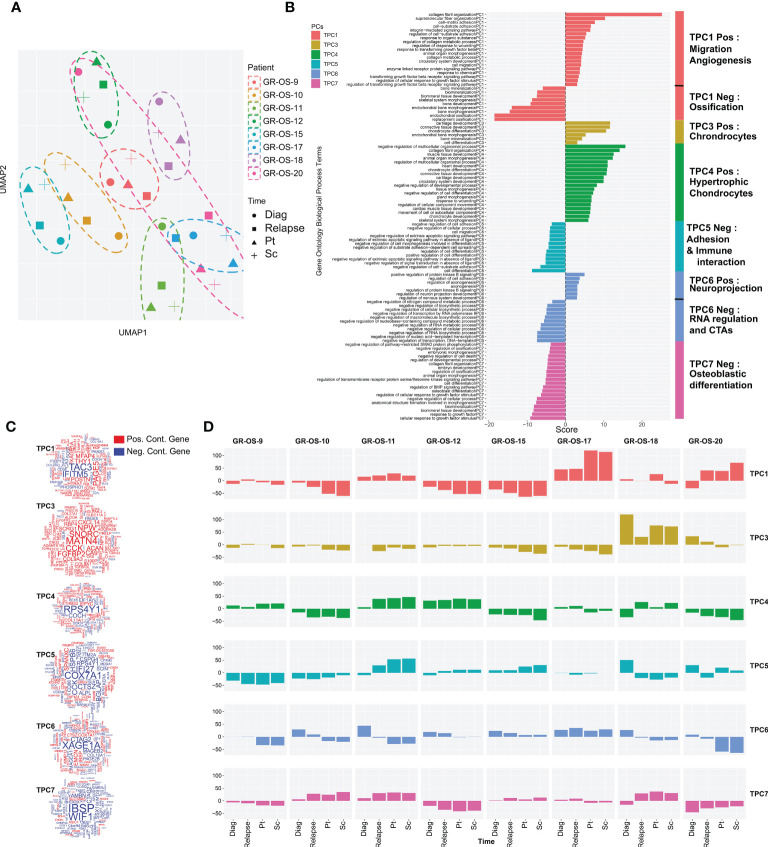
Tumor transcriptomic comparison between osteosarcoma PDX models and corresponding human samples at relapse and diagnosis. **(A)** UMAP of the selected tumor principal components (TPCs) for the tumor fraction; **(B)** Geneset enrichment analysis of each component based on gene contribution to the TPC; The sign of the enrichment score represents the opposite functional enrichment described by the same principal component **(C)** A word cloud illustrating the gene contributing the most to each component, negatively (blue) or positively (red); **(D)** The contribution of each sample to each TPC classified by patient. The sign of the contribution illustrates if the sample participates in the negative functional enrichment fraction of the corresponding principal component shown in **(C)**. Diag, diagnosis; pt, paratibial; sc, subcutaneous.

Osteosarcoma tumors present great plasticity due to their ability to spread to other organs and resist chemotherapy regimens. Tumor grafting in mice constitutes a new microenvironment (ME) to colonize, which could, similar to metastatic dissemination, drastically alter the transcriptomic program of tumors and preclude their preclinical significance. To address this question, we first classified tumors according to their tumoral transcriptome in the principal component space. Genes exclusively expressed in human tumors but not in the human fraction of the PDX immunodeficient models were filtered out to exclude genes expressed by the ME and therefore focus our comparison on tumor cell expression (**Supplementary Figures S4A–G**, cf. *Methods*). A total of 921 genes were thus removed from our comparison (**Supplementary Table S3**), and a functional enrichment analysis of the removed genes confirmed, as expected, their association with the tumor microenvironment (TME) and the immune infiltrate (**Supplementary Figure S4F**; **Supplementary Table S3**). We then selected the first six principal components that together explain 54% of the gene expression variance of tumor cells. We call them tumor principal components (TPCs). Uniform Manifold Approximation and Projection (UMAP) of the TPCs shows that apart from GR-OS-20_Pat_D (the diagnostic sample), all the samples from the same patient or derived PDX models clustered together at the transcriptomic level (**Figure 3A**; **Supplementary Figure S4H**). This result suggests that for six out of seven patients that the tumor transcriptome does not vary more than the inter-patient variance through disease progression or even xenograft. The GR-OS-20 exception is probably explained by the late relapse in this patient, with 5 years between the diagnosis and relapse. All six TPCs are associated with well-described pathways in osteosarcoma corresponding either to histological subtypes (TPC3: chondrocytes, TPC4: hypertrophic chondrocytes, TPC6: mesenchymal/cancer testis antigen, TPC7: osteoblast differentiation), tumor cell activity (TPC1: ossification/migration/angiogenesis), or tumor interaction with ME (TPC5: adhesion/immune interaction) (**Figures 3B–D**; **Supplementary Table S3**). At diagnosis, relapse, and in the PDX models, the pathways expressed in the tumor generally concorded with the histopathological description of the primary tumor in the PDX models (**Figure 3D**). Thus, TPC1 confirmed at the transcriptional level the higher calcification observed in GR-OS-10, GR-OS-12, and GR-OS-15 and showed a proximity between GR-OS-17 and GR-OS-20, potentially explained by their common preponderant giant cell phenotypes. Likewise, TPC3 captured the chondrocytic transcriptional program related to the chondroblastic subtype of the GR-OS-18 model, which apparently preexisted in the patient at diagnosis. GR-OS-11 and GR-OS-12, both described histologically as mixed osteoblastic and chondrocytic cells, expressed genes involved in the formation of hypertrophic chondroblasts (TPC4) known to transdifferentiate into osteoblasts and/or osteocytes (20). The three other TPCs described respectively adhesion/immune interaction (TPC5), expression of specific cancer testis antigens (CTA, TCP6), and osteoblastic differentiation (TCP7). Although interesting and often conserved longitudinally from patient to model, none of these gene expressions or biological functions were detectable at the histological level and therefore comparable in this study. To validate our observations at the protein level, we submitted frozen samples from the eight paratibial PDX models to mass spectrometry. We again used principal component analysis to decipher the proteomic profiles of each sample (**Figure S5**). This analysis supported our observation at the transcriptomic level about the proximity between GR-OS 12, GR-OS-15, and GR-OS-10 (**Figures S5A, B**) with a common calcification program (**Figure S5C**, PC1). Strikingly, PC1 and PC3 (**Figure S5E**), as well as the full proteomic profile (**Figure S5B**), suggested a high similarity of both osteosarcomatosis (**Figure S5B**) with a proliferative phenotype emphasized by the contribution of the MCM complex proteins, MCM6 and MCM5, to the PC3.

### Cross-species comparison of tumor microenvironment shows similarities between bone microenvironment in osteosarcoma PDX models and patient tumors

Osteosarcoma cells maintain several dependencies on the TME, specifically osteoclasts. Several publications recently described strong links between TME composition and overall and event-free survival (11). PDX models were developed in immunodeficient NSG mice, which lack mature T cells, B cells, and natural killer cells and have many defects in innate immunity (21, 22). Neutrophils and monocytes should constitute most of the remaining mouse immune cells detectable in peripheral blood. Dysfunctional dendritic cells and macrophages are also present in these mice.

**Figure 4:**
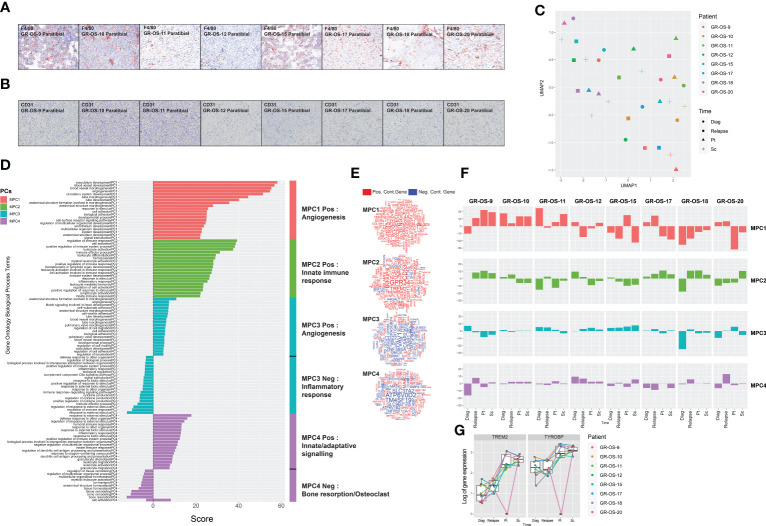
Tumor microenvironment composition comparison between osteosarcoma PDX models and corresponding human samples at relapse and diagnosis. **(A)** Identification of F4/80^+^ cells in eight paratibial PDX models of osteosarcoma; **(B)** Identification of CD31 cells in eight paratibial PDX models of osteosarcoma; **(C)** UMAP of the four first microenvironment principal components (MPCs); **(D)** Geneset enrichment analysis of each component according to genes positively or negatively contributing to MPC; The sign of the enrichment score represents the opposite functional enrichment described by the same principal component **(E)** A word cloud illustrating the gene contributing the most to each component, negatively (blue) or positively (red); **(F)** Contribution of each sample to each MPC classified by patient. The sign of the contribution illustrates if the sample contributes to the negative functional enrichment fraction of the corresponding principal component shown in **(D)** Diag, diagnosis; pt, paratibial; sc, subcutaneous. **(G)** Boxplot showing the log-transformed of the gene expression of TREM2 and TYROBP at diagnosis, relapse, and in the mouse fraction of the paratibal and subcutaneous PDX models.

We observed osteocondensation and osteolysis with CT scan and HES (hematoxilin–eosin–safranin) in PDX models, and PDX TME seems composed of a variety of osteoclast, macrophage, endothelial, and fibroblastic cells. F4/80 immunostaining confirmed the presence of murine macrophage infiltrates in all paratibial models (**Figure 4A**; **Supplementary Figures S6A–E**). The CD31 immunostaining revealed a unique vascular phenotype shared by all models of tumor vessels distributed throughout the tumor (**Figure 4B**). We further explored mouse TME in sc- and pt-PDX models and compared it to patient TME at the transcriptomic level using RNA-Seq. To do so, using the cross-species nature of xenografted tissues, we extracted genes that were exclusively expressed in the patient samples and not in the human fraction of the PDX, considering them purely microenvironmental. We then selected the orthologous genes in the mouse fraction of the PDX and merged them after normalization in a cross-species gene expression matrix to compare patient and murine TMEs (**Supplementary Figures S6A–E**, cf. *Methods* for detail). This comparison, although imperfect due to several biases related to the bulk nature of the RNA-Seq, suggests that some models share a common TME composition with the corresponding patient samples. Similarly to the previous section, we had to work in the principal component space to reduce the dimensionality of the data and called the resulting components microenvironmental principal components (MPCs). UMAP of the MPCs (**Figure 4C**) suggests that GR-OS-9, GR-OS-10, GR-OS-15, and GR-OS-18 models present the most conservative TME between human and PDX samples with similar levels of vascularization (MPC1, **Figures 4D–F**) or immune infiltrates (MPC2, **Figures 4D–F**).

The first MPC divided the sample by vascularization while MPC2 clearly defined a group of tumors with distinct immune infiltrates (**Figures 4D–F**). Strikingly, the best contributor genes to MPC2 involved *TREM2/TYROBP* (**Figure 4E**), an axis associated with immunosuppression (23), suggesting that MPC2 might correspond to tumor associated macrophage (TAM) infiltrates despite the immunodeficiency of NSG mice. The TREM2/TYROBP complex was also detected at the gene expression level (**Figure 4G**), suggesting a conserved high infiltration by TAM in GR-OS-9, GR-OS-17, and GR-OS-18. MPC3 and MPC4 were mostly explained by the extreme variance in the two samples (**Figure 4F**).

## Discussion

In this work, we drew a multimodal portrait of newly established osteosarcoma subcutaneous and paratibial/orthotopic PDX models in immunodeficient NGS mice. Originating from highly refractory relapsed tumor samples of adolescents and young adults, the non-dissociated tumor fragments transplanted grow at both subcutaneous and paratibial sites and mimic the morphologic, genetic, and transcriptomic features of the tumor of origin.

Few osteosarcoma PDX models have yet been described (12, 14), all from dissociated patient-derived tumor cells injected subcutaneously and more rarely in an orthotopic intraosseous setting (14). Here, we implanted tumor pieces without previous dissociation, both subcutaneously and paratibially, after periosteum denudation. Tumor piece implantation has the advantage of preserving the 3D structure of the tumor and may decrease the risk of direct tumor cell passage to the circulation, as observed with injections of dissociated cells (24). Nevertheless, unusual spleen metastases were still observed in nearly all orthotopic PDX models.

As expected, once established (P2 passage), sc-PDX were easy to implant, had a 100% tumor take rate, were easy to monitor locally (and noninvasively), displayed growth rates compatible with drug testing, and had nearly no metastatic spread. Conversely, pt-PDX harbored a local and metastatic behavior closer to that observed in patients, with three out of the eight pt-PDX models having metastatic spread to the lungs, bones, and liver.

As previously observed in other models, osteosarcoma PDX closely recapitulates the genomic landscape of its originating relapse sample. Driving mutations/deletions and oncogene amplifications present in the relapse sample were present in the corresponding PDX models, as usually reported (14, 25). We also highlighted the importance of analyzing samples from diagnosis and across time to reveal key drivers and attempt to distinguish abnormalities involved in oncogenesis from those acquired or selected during the relapse, metastatic, or refractory process. Both could be further explored as therapeutic targets.

**Figure 5:**
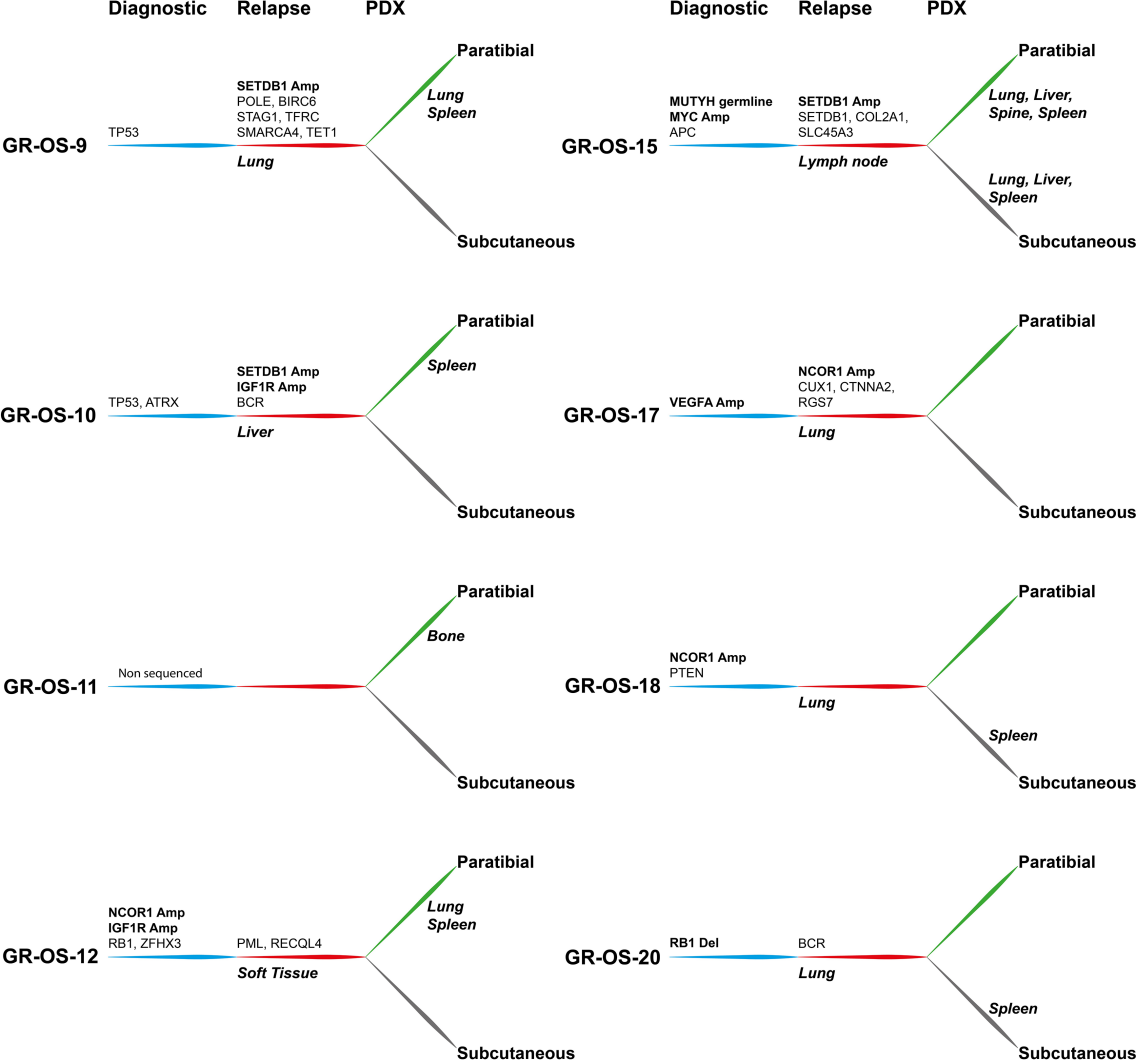
Tumor alterations and metastatic development for each patient at diagnosis and at relapse and for their derived subcutaneous and paratibial PDX models. All depicted alterations were conserved from either diagnosis (blue) or relapse (red) to PDX models (green and gray for paratibial and subcutaneous, respectively).

Some of our osteosarcoma PDX models present an amplification of *MYC* (GR-OS-15), *VEGFRA* (GR-OS-17), or a pathogenic variant in *PTEN* (GR-OS-18), for which Genome-Informed Targeting has already been proven with CDK9 inhibitors, multikinase inhibitors or anti-*VEGF*, and *AKT* or *mTOR* inhibitors, respectively (12). Our models complete those published by Sayles (12) with two models harboring amplification of *IGF1R* (GR-OS-10, focal in GR-OS-12, **Figure 5**), observed in two out of 129 osteosarcoma patients at diagnosis (http://www.cbioportal.org/study/summary?id=sarcoma_mskcc_2022) and five at relapse (MAPPYACTS), targetable by anti-*IGFR* antibodies. *TP53* mutations (GR-OS-9 and GR-OS-10) might also suggest *WEE1* inhibitor use (26). Another model (GR-OS-17) presents a fusion involving the *TP53* gene. These different models might be used to test and analyze *WEE1* inhibitor efficacy according to *TP53* alteration type. Several clinical trials are ongoing either in osteosarcoma regardless of tumor genetic *TP53* alteration (e.g., NCT04833582) or molecularly driven in pediatric cancer with selected *TP53* alterations (e.g., AcSé-ESMART Arm C, NCT02813135).

Along with driver genetic abnormalities at diagnosis, of which several were already described at diagnosis in other cohorts (27), new abnormalities emerging at relapse might be interesting to target osteosarcoma metastatic potential. Most of these genes are involved in DNA repair/modification processes (GO:0006259; *TRRAP*, *SETDB1*, *POLE*, *TET1*, *RECQL4*, *PML*, *TFRC*); chromosome organization (GO:0051276; *STAG1*, *SMARCA4*, *TRRAP*, *SETDB1*, *POLE*, *TET1*, *RECQL4*, *PML*); and cell cycle process (GO:0022402: *STAG1*, *TRRAP*, *BIRC6*, *POLE*, *RECQL4*, *PML*), which might provide clues about mechanisms of tumor cell evasion from tumor suppressors, therapy, or the patient’s immune response.

Epigenetic modulation is a promising therapeutic field in osteosarcoma. Relative genomic hypomethylation was shown to be strongly predictive of response to standard chemotherapy (8). Recurrence and survival were associated with genomic methylation, but through more site-specific patterns (8). We found that in our patients and their derived models, *NCOR1* (histone desacethylase complex) and *SETDB1* (28) (histone methyltransferase that specifically trimethylates ‘Lys-9’ of histone H3) were amplified in a mutually exclusive manner. In patients, *SETDB1* amplification was detected only at relapse, with one model presenting an acquired additional *SETDB1* mutation, while *NCOR1* amplification could be detected at both diagnosis and relapse, according to the models. The role of these amplifications in histone regulatory genes and their therapeutic value remain to be explored. The *SETDB1-TRIM28-ZNF274* complex may play a role in recruiting *ATRX* to the 3’-exons of zinc-finger coding genes with atypical chromatin signatures to establish or maintain/protect H3K9me3 in these transcriptionally active regions (29). One patient had an *ATRX* mutation (heterozygotic, Stop Gain) at diagnosis and *SETDB1* amplification at relapse (GR-OS-10). Between 13% and 47% of high-grade osteosarcomas have been found to contain amplification of several genes that map to a region of chromosome 17p11.2, including COP9 constitutive photomorphogenic homolog subunit 3 (*COPS3*), nuclear receptor corepressor (*NCOR1*), target of myb1-like 2 (*TOM1L2*), and peripheral myelin protein 22 (*PMP22*), and may be involved in osteosarcoma tumorigenesis (30). Our osteosarcoma PDX models might be interesting to further explore these pathways functionally and as therapeutic options.

The transcriptomic landscape of these models was studied mostly to confirm the expression relevance of genomic amplifications and for fusion detection. We showed that PDX models not only conserved the genomic landscape of the originating patient tumor but also conserved several characteristics of the tumor transcriptomic programs identified in human tumors at diagnosis or relapse, which reinforces their preclinical significance.

To analyze the omic resources gathered in this study, we developed a pipeline, available on GitHub (PDXploreR), taking advantage of the cross-species’ nature of PDX models to deconvolute the tumor cells from the TME. Applied to drug testing, this strategy should improve our understanding of the drug’s impact on both cell populations separately and therefore facilitate the identification of appropriate biomarkers. For instance, we anticipate a better capture of the broad activity of multi-kinase inhibitors on both tumor and vessel cells.

## Conclusion

Beyond similarity, the clustering of PDX models at both the genomic (WES) and transcriptomic (RNA-Seq) levels with the matched human samples attests to high inter-patient heterogeneity. This heterogeneity underlies the necessity to generate more PDX models to shed light on the complexity of osteosarcoma. The ongoing work of the Pediatric Preclinical Proof of Concept Platform from the Innovative Therapies for Children with Cancer consortium (ITCC-P4, www.itccp4.eu) and other similar programs (e.g., NCI PPTC, www.ncipptc.org) by increasing the number of PDX models should allow more accurate preclinical drug testing and help define the corresponding biomarker of efficacy/resistance. This goal will be facilitated by new techniques in single cell and spatial transcriptomics that are more easily applicable to PDX models due to the availability of tumor material.

## Methods

### Translational research context and patients’ characteristics

The MAPPYACTS clinical trial (Molecular Profiling for Pediatric and Young Adult Cancer Treatment Stratification) was a prospective, multicentric, clinical proof-of-concept study to stratify targeted therapies adapted to molecular profiling of relapsed and refractory pediatric tumors (17, 31). Ancillary studies included the development and characterization of patient-derived xenograft (PDX) models and primary cell lines (manuscript in preparation). Eight osteosarcoma PDX models were established at Gustave Roussy from two girls and six boys, aged 13 to 20 years old (**Supplementary Figure S1**; **Supplementary Table S1**) at the time of inclusion. Six patients presented with early (5 to 30 months from diagnosis) first (n = 2) and second (n = 4) relapses and two late first (M55 for GR-OS-20) and fourth (M11 for GR-OS-17) relapses. All had metastatic disease either in the lung (n = 2) or at multiple sites (n = 6), and one had an additional loco-regional relapse. The tumor biopsy/surgery samples used were from lesions located in the lung (n = 3), liver (GR-OS-10), lymph node (n = 3), and intramuscular (GR-OS-18) sites. A range of initial histological sub-types were represented at diagnosis (osteoblastic n = 4, fibroblastic GR-OS-11_Pat_D, chondroblastic GR-OS-15_Pat-D, telangiectatic GR-OS-10_Pat-D, and giant cell GR-OS-17_Pat-D). All patients presented aggressive osteosarcoma from diagnosis: two had a rare presentation of osteosarcomatosis at diagnosis (GR-OS-12_Pat-D, GR-OS-15_Pat-D), three had initial metastatic disease, three progressed under chemotherapy (at week 7: GR-OS-10_Pat-D, GR-OS-15_Pat-D; at M5: GR-OS-12_Pat-D), and four had poor histological responses to neoadjuvant chemotherapy. All patients were heavily treated before sample collection and had received the five major drugs used in osteosarcoma (methotrexate, doxorubicin, cisplatin, ifosfamide, and etoposide), except one patient who did not receive methotrexate (GR-OS-12_Pat-D). All patients but one died of progressive disease, with a median delay of 24 months (range 9–124 months) from initial diagnosis and 10 months (range 1–19) from relapse used for MAPPYACTS studies. The last patient was alive in second complete remission two years after a late isolated lung relapse (at 111 months), treated by surgery alone.

### Human refractory/relapsed osteosarcoma tumor sample collection

Following informed consent, tumor samples were collected by surgical resection, CT, or ultrasound-guided intentional tumor biopsy. One piece of tumor was immediately snap frozen for clinical sequencing analysis, and a fresh one obtained at the same time was immediately placed in transport media (DMEM with 1% antibiotics), conserved at 4°C for a maximum of 48 h, immediately transferred to the research laboratory at room temperature, or soft frozen in fetal bovine serum (FBS) containing 10% DMSO (dimethyl sulfoxide) preserved at −80 °C.

Clinical patient analysis (WES and RNA-Seq) and data interpretation were performed as described by Berlanga (17).

For the development of preclinical models, the samples were immediately processed upon arrival at Gustave Roussy, as described below.

Snap frozen tumor samples at diagnosis issued from the patients with successful PDX models were collected and analyzed with the same techniques (WES, RNA-Seq).

### *In vivo* orthotopic human osteosarcoma PDX models development

Experiments were validated by the CEEA26 Ethic Committee (approval numbers: 2015032614359689 v7 and 201912111337397 v2) and carried out under conditions established by the European Community (Directive 2010/63/UE). Animals were purchased at Gustave Roussy (Villejuif, France) and maintained in the respective animal facilities following standard animal regulations, health and care, and ethical controls.

Under anesthesia (3% induction, 2% maintenance isoflurane, and 1.5 L/min air), 21 tumor samples were directly implanted without previous cell dissociation either subcutaneously (~5 mm^3^; n = 12) by performing a skin incision on the mouse back and placing the tumor sample under the skin (32) in both flanks and/or paratibially (~2 mm^3^, n = 16), on orthotopic position, between muscle and bone tibia after a 0.5 cm skin incision and a gentle activation of the periosteum (periosteum denudation) (33), in immunocompromised NOD.Cg-Prkdc^scid^IL2rg^tm1Wjl^/SzJ (NSG) mice. To avoid bone pain, an analgesic (buprenorphine at 0.3 mg/kg) was applied in addition to general anesthesia or when symptoms appeared. Clinical status, tumor uptake, and tumor growth were evaluated one to three times a week. Subcutaneous and paratibial xenografts were detected by palpation, tumor gross apparition (caliper measurements), as well as bone structure alterations by CT scan imaging for paratibial models. The experiments lasted until tumors reached specific endpoints (significant weight loss, difficulty walking). Mice were sacrificed when tumor volume reached around 1,500 mm^3^ subcutaneously or when clinical signals (e.g., difficulties moving) appeared in paratibial models. Then for each further passage, the PDX tumor sample was divided into several pieces: for new mouse implantation (subcutaneous and/or paratibial), soft freezing (frozen in FBS + 10% (v/v) DMSO), snap freezing (frozen in nitrogen), and for histology (paraffin embedded) (**Figure 1A**).

Tumor samples from patients with recurrent osteosarcoma were transplanted both subcutaneously and paratibially into NSG mice.

Tumor doubling time (Td) was determined in an exponential growth phase between 200 and 400 mm^3^, for the subcutaneous models (31).

### *In vivo* CT scan imaging

IVIS SpectrumCT (Perkin Elmer, Courtaboeuf, France) was used for image acquirement. This system allows the primary tumor and metastases to be detected by X-ray tomography co-registered with optical images. The lower section of the mouse body (area of the lower legs) was imaged for primary tumor detection and the chest for metastatic spread to the lungs. CT scan imaging was performed under anesthesia with 3% (v/v) isoflurane.

### Histology and immunohistochemistry

Organs were fixed in 4% (v/v) paraformaldehyde and embedded in paraffin. Tissues were stained with hematoxilin–eosin–safranin (HES) for morphology or processed for IHC. Briefly, after dewaxing and rehydration, tissue sections were submitted to heat-induced antigen retrieval (ER2-corresponding EDTA buffer pH 9) for 20 min at 100°C. Slides were incubated with the following primary antibodies for 1 h at room temperature: mouse monoclonal anti-human Ki67 antibody (clone MIB1; 1:20; Agilent Dako), anti-F4/80 (Cell Signaling Ref 70076 clone D2S9R (1/1500)), and anti-CD31 polyclonal (Abcam Ref: ab28364, (1/50)). The nuclear signal was revealed with the Klear mouse kit (GBI Labs). Slides were examined using light microscopy (Zeiss, Marly-Le-Roy, France), and a single representative whole tumor tissue section from each animal was digitized using a slide scanner NanoZoomer 2.0-HT (C9600-13, Hamamatsu Photonics). Histology was reviewed by a bone-expert pathologist.

### *In vitro* primary and secondary cell culture

Osteosarcoma cells from human osteosarcoma relapsed samples were cultured *in vitro* directly from the patient tumor sample (primary cultures) or from established (at least P2) osteosarcoma PDX models (secondary PDX-derived cell cultures). For both types of cultures, the tumor sample was cut into several small pieces and dissociated mechanically with a 22-gauge needle in medium. The tumor preparation was resuspended in Dulbecco’s modified Eagle medium (DMEM, GIBCO/Invitrogen, Saint Aubin, France), supplemented with 20% (v/v) fetal bovine serum (FBS, GIBCO/Invitrogen, Saint Aubin, France), plated in T75 flasks, and incubated at 37°C in a humidified atmosphere (5% CO_2_ and 95% air). All the procedures were performed under sterile conditions. Mycoplasma tests were performed each month by PCR. All attempts at primary cell culture failed. Only the results of secondary PDX-derived cell culture are presented.

### Compounds

The compounds doxorubicin, methotrexate, cisplatin, and etoposide were purchased from Sigma Aldrich (St. Louis, MO, USA), mafosfamide from Toronto Research Chemicals Inc. (TRC) (Toronto, Canada), and regorafenib, pazopanib, and cabozantinib from LC Laboratories (US, Canada). All the compounds were diluted in dimethyl sulfoxide (DMSO) (Sigma Aldrich, St. Louis, MO, USA), except cisplatin, which was diluted in N,N-dimethylformamide (DMF) (Sigma Aldrich, St. Louis, MO, USA), and stored at −20°C in a 10 mM stock solution.

### Treatment (MTS assay)

Growth inhibition was determined using the CellTiter 96 Aqueous One Solution Cell Proliferation Assay (MTS assay) (Promega Corporation, Charbonnieres, France), according to the manufacturer’s instructions and as performed before (33).

Cells from PDX secondary cultures were seeded at 7,000 cells/well in a 96-well plate as described before and incubated at 37°C overnight. Cells were treated with doxorubicin, MTX, etoposide, mafosfamide, cabozantinib, regorafenib, or pazopanib at concentrations ranging from 0 to 100 μmol/l, or with cisplatin at 0 to 50 μmol/l. Seventy-two hours later, cell viability was determined by adding 20 μl of MTS solution to each well and measuring (490 nm) 1–5 h after incubation at 37 °C in an automatic plate reader (Elx808; Fisher Bioblock Scientific SAS, Illkirch, France). The IC50 was calculated as the drug concentration that inhibits cell growth by 50% compared with control.

### Molecular characterization of human samples and *in vivo* PDX models (WES, RNA-Seq)

Human osteosarcoma patient samples and PDX samples, either subcutaneous (Passage 2 for all models) or paratibial (Passage 1 for GR-OS-9, GR-OS-10, and GR-OS-12 and Passage 2 for GR-OS-15, GR-OS-17, GR-OS-18, and GR-OS-20), were snap frozen in liquid nitrogen and stored at −80°C until extraction. Tumor DNA and RNA and germline DNA were isolated using the AllPrep DNA/RNA micro kit (Qiagen, Germany) according to the manufacturer’s instructions.

Whole exome (WES) and RNA sequencing analyses were performed as previously described (34). Whole exome sequencing (WES) was performed on 500 ng of tumor tissue using Agilent SureSelect V5 (50Mb) or Clinical Research Exome (54 Mb) kits. RNA sequencing libraries were prepared with the TruSeq Stranded mRNA kit following recommendations: the key steps consist of PolyA mRNA capture with oligo dT beads (1 µg total RNA), fragmentation to approximately 400 pb, DNA double strand synthesis, and ligation of Illumina adaptors for amplification of the library by PCR for sequencing. Libraries sequencing was performed using Illumina sequencers (NextSeq 500 or Hiseq 2000/2500/4000) in 75 bp paired-end mode in both techniques, and data sequencing was processed by bioinformatics analyses.

### Mass spectrometry analysis (proteomics)

*Sample preparation*: Cryo-conserved PDX samples were lysed at room temperature in 8M urea and 50 mM ammonium bicarbonate. Lysates were then sonicated and centrifuged at 20,000×*g* for 10 min. Following protein quantification by BCA, extracted proteins were reduced by adding 5 mM DTT at 55°C for 30 min, then subsequently alkylated by adding 10 mM iodoacetamide at room temperature in the dark for 30 min. Samples were diluted 10-fold with 50 mM ammonium bicarbonate buffer, reducing the urea concentration below 1 M, before an overnight digestion with trypsin at a 1:50 enzyme:protein ratio at 37°C. The aliquots were acidified by the addition of *TFA* to a final concentration of 1% (v/v) for 15 min at 4°C and centrifuged at 2,000×*g* for 10 min to remove precipitates. Digested extracts were loaded onto C18 desalting columns (Waters, Sep-Pak Vac RC; 50mg sorbent WAT054955) previously equilibrated with 0.1% TFA. Peptides retained on the C18 column were washed three times with 0.1% TFA, then with 0.1% TFA + 5% acetonitrile, before elution using 600 µl of 0.1% TFA + 40% acetonitrile.

*LC–MS/MS analysis:* Online chromatography was performed with an RSLCnano system (Ultimate 3000, Thermo Scientific) coupled online to an Orbitrap Exploris 480 mass spectrometer (Thermo Scientific). Peptides were trapped on a C18 column (75 μm inner diameter × 2 cm; nanoViper Acclaim PepMapTM 100, Thermo Scientific) with buffer A (2/98 MeCN/H2O in 0.1% formic acid) at a flow rate of 3.0 µl/min over 4 min. Separation was performed on a 50 cm × 75 μm C18 column (nanoViper Acclaim PepMapTM RSLC, 2 μm, 100Å, Thermo Scientific) regulated to a temperature of 40°C with a linear gradient of 3% to 32% buffer B (100% MeCN in 0.1% formic acid) at a flow rate of 300 nl/min over 211 min. MS full scans were performed in the ultrahigh-field Orbitrap mass analyzer in the ranges m/z 375–1500 with a resolution of 120,000 at m/z 200. For every full scan, the top 20 most intense ions were isolated and subjected to further fragmentation *via* high-energy collision dissociation (HCD) activation at a resolution of 15,000 with the AGC target set to 100%. We selected ions with charge states ranging from 2+ to 6+ for screening. Normalized collision energy was set at 30 and the dynamic exclusion at 40 s.

*Data pre-processing*: For identification without the contribution from the host species, the data were searched against both *Homo sapiens* (UP000005640) and *Mus musculus* (022017) UniProt databases using Sequest HT through Proteome Discoverer (version 2.4) and then keeping the proteotypic peptides to human sequence for protein quantification. Enzyme specificity was set to trypsin, and a maximum of two miss-cleavage sites were allowed. Oxidized methionine, met-loss, met-loss-acetyl, and N-terminal acetylation were set as variable modifications. The carbamidomethylation of cysteins was set as a fixed modification. The maximum allowed mass deviation was set to 10 ppm for monoisotopic precursor ions and 0.02 Da for MS/MS peaks. The resulting files were further processed using myProMS v3.9.3 (35). The FDR calculation used Percolator and was set to 1% at the peptide level for the whole study. The label-free quantification was performed by peptide-extracted ion chromatograms (XICs) computed with MassChroQ version 2.2.21. For protein quantification, XICs from all proteotypic peptides were used, and missed cleavages were allowed. Median correction and variance scale normalization were applied to the total signal to correct the XICs for each PDX. LFQ was performed following the algorithm as described (36), with the minimum number of peptide ratios set to 1 and the large ratio stabilization feature, and the LFQ values were also normalized to correct for remaining total intensity biases. The final LFQ intensities were used as protein abundance, with 3,547 proteins identified specific to the human genome and 22.4% missing values. Before performing any downstream analysis, missing values were imputed using the principal component analysis (PCA) function (37). Only those proteins with a missing rate less than 34% missing values were imputed. After imputation, the number of proteins deemed valid for downstream analyses was boosted to 2,509 proteins.

### Molecular comparison of human samples and *in vivo* PDX-models (WES and RNA-Seq)

RNA-Seq and WES of eight tumor patient samples at diagnosis and relapse and from subcutaneous and paratibial (orthotopic) PDX models were analyzed. Samples with a RIN lower than six were removed from the study (GR-OS-18-Pat_D).

Human and mouse sequences were discriminated using Xenome (38) in PDX samples. This tool creates a chimeric index from the two reference genomes of the graft and host species. The genome versions hg19 and mm10 were used for the graft and host, respectively. Once the index is computed, each fastq file is separated by Xenome into five new fastq files. The output fastq files corresponding was kept, without ambiguity, to the graft or the host genome or transcriptome. Corresponding R1 and R2 fastq files were obtained using the option paired in the Xenome tool.

After human fraction isolation from the mouse environment of xenograft samples, RNA-Seq and WES data were analyzed. For the whole exome, graft fastq files were used, and the alignment of the fastq files with the reference genomes was performed. For that, a pipeline was created (ref: https://github.com/Rdroit/PDXploR) using the BWA best practices to produce the BAM and Pileup files. An in-house script was used to compute the variant call with Varscan2 (39). The variants are annotated with Annovar (40). Somatic alterations with less than five minimum reads supporting the mutations, 5% of the reads covering the sequence supporting the alteration, and more than 1% of the population annotated with the mutation in the databases 1000g2015aug (latest 1,000 Genomes Project dataset with allele frequencies in six populations including ALL, African, Admixed American, East Asian, European, and South Asian) and kaviar_20150923 (latest Kaviar database with 170 million variants from 13K genomes and 64K exomes) were filtered out. Sample similarity estimation based on the somatic mutations was performed using Jaccard distance.

This measures the dissimilarity between two ensembles. The higher the Jaccard distance, the farther the ensembles are. Using this tool, all the different types of samples are compared.

Copy number variation (CNV) profile estimation was obtained using the pileup files by Facets (41) analysis. The different categories of CNV considered were amplification (≥7 copies), focal amplification (≥7 copies on less than 5 Mb), gain (≥2 and <7), and loss (=1), Deletion (=0). Jaccard distance allowed the comparison of every type of sample with others based on the ensembles of copy number alterations.

In RNA-Seq, in PDX models, the human fraction represents the gene expression profile of tumor cells, while the mouse fraction corresponds to the gene expression profile of mouse TME. PDX model and patient tumor expression comparison was obtained by isolating the tumor cell expression program from the ME expression program using Xenome (37). The Xenome program uses a meta-reference genome created with the two species reference genomes (here hg19 and mm10) to separate the files between host and graft sequences. The fastq files obtained were then aligned using Salmon (42) on the reference transcriptomes of hg19 for graft fastqs and mm10 for host fastqs. The PDX human fraction was used to isolate the tumor expression in the human samples using the differential expression package Deseq2 (43). A functional enrichment was then performed on the 921 removed genes (confirmation of the correct microenvironment fraction from the patient sample removal).

The remaining genes represent the tumor. Principal component analysis was used to decompose the expression of the tumor cells. A selection of the components was then performed based on their contribution to global variance. A component was kept if it contributed to more than 5% of the overall variance and if it did not have a significant human/mouse sample separation effect. A clusterization was performed using the most contributing components (to verify if patient and PDX samples were grouped by patient).

Each component is then functionally enriched for genes representing the maximum variance. To validate this analysis and see the link between phenotype observations and expression decomposition, those enrichments were compared to the previous cell composition analysis.

The ME is known to have great importance in osteosarcoma. To estimate the reproducibility of the xenograft, it is necessary to isolate and compare the expression of the patient and PDX ME. The mouse-associated sequences removed by Xenome represent the microenvironment in the mouse. To estimate the similarity between the human and mouse ME, the removed expression in the xenograft samples is compared to the expression removed by the differential analysis in the patient samples. The expression profiles are scaled by the nature of the samples. Then a PCA is performed to identify the composition of those profiles and their conservation over time. Common genes removed between the two groups are conserved. Functional enrichment was performed on the genes that were not present in mice (<3 reads per sample), revealing most immunity components. Also, an enrichment on the common genes (>3 reads per sample) was done, revealing the common ME composition. Then, the ME composition was decomposed with PCA, and each significant component was then enriched to be identified.

The expression of fusion transcripts in RNA-Seq data was performed using the nf-core pipeline Fusions (https://github.com/nf-core/rnafusion) with a first call using the tools Arriba 1.2.0, EricScript 0.5.5, Pizzly 0.37.3, Squid 1.5, and Star Fusion 1.8.1 (44–48), all with the reference transcriptome of hg19. The results were then filtered, and only the ones found by at least three of the five different tools were kept. To validate those conserved fusions, the tool Fusion Inspector 2.2.1 was used to search for the selected fusions in all the samples (49). Finally, a fusion was considered real when it was found by Fusion Inspector with at least one read covering the breakpoint between the two genes. The resulting fusions were then annotated in different categories based on the type of alteration: 3’ altered, 5’ altered, and truncated.

### Circos construction

To integrate somatic mutations, copy number alterations, gene expression, and RNA fusions over time points, we generated, using the package Rcircos (50), a circos summarizing all this data for each patient.

### Statistical analysis

The data were shown as the mean ± standard error of mean (SEM) using Graphpad Prism ^®^ Software version 9.00 (Graphpad Software Inc., La Jolia, CA, USA).

## Data availability statement

The datasets presented in this study can be found in online repositories and are available upon request from the authors through the BoOSTDataS data access committee (boostdatas.org).

## Ethics statement

The studies involving human participants were reviewed and approved by CEEA26, Ethic committee (approval number: 2015032614359689 v7 and 201912111337397 v2). Written informed consent to participate in this study was provided by the participants’ legal guardian/next of kin. The animal study was reviewed and approved by CEEA26, Ethic committee (approval number: 2015032614359689 v7 and 201912111337397 v2. Written informed consent was obtained from the minor(s)’ legal guardian/next of kin for the publication of any potentially identifiable images or data included in this article.

## Author contributions

Conception and design: MdC, NG, and BG. Development of methodology: MdC, NG, RD, AM, and BG. Acquisition of data: MdC and BG. Analysis and interpretation of data: MdC, NG, AG-B, PK, RD, AM, and BG. Writing of the manuscript: MdC, RD, NG, AM, and BG. Study supervision: NG and AM. All authors contributed to the article and approved the submitted version.
