# Supplementary material for: Longitudinal characterization of primary osteosarcoma and derived subcutaneous and orthotopic relapsed patient-derived xenograft models
Source: Front Oncol. 2023 Jun 12;13:1166063. doi: 10.3389/fonc.2023.1166063 (PMC10291137; doi:10.3389/fonc.2023.1166063)
Supplement: Supplementary file 1 [file DataSheet_1.pdf]

A

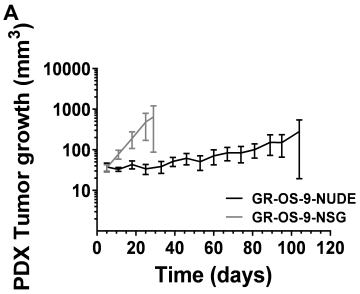

B

PDX_sc	Strain type	First tumor detection median time (days)	Median time Detection-Sacrifice (days)	Tumor aspect
GR-OS-9	Nude	67	32,5	calcified
GR-OS-9	NSG	11	18	calcified

Supplementary Fig.S1: Tumor growth characteristics of subcutaneous implantation in Nude and NSG mice for PDX GR-OS-9. **A.** Tumor growth in Nude versus NSG with the time; **B.** Tumor development characteristics

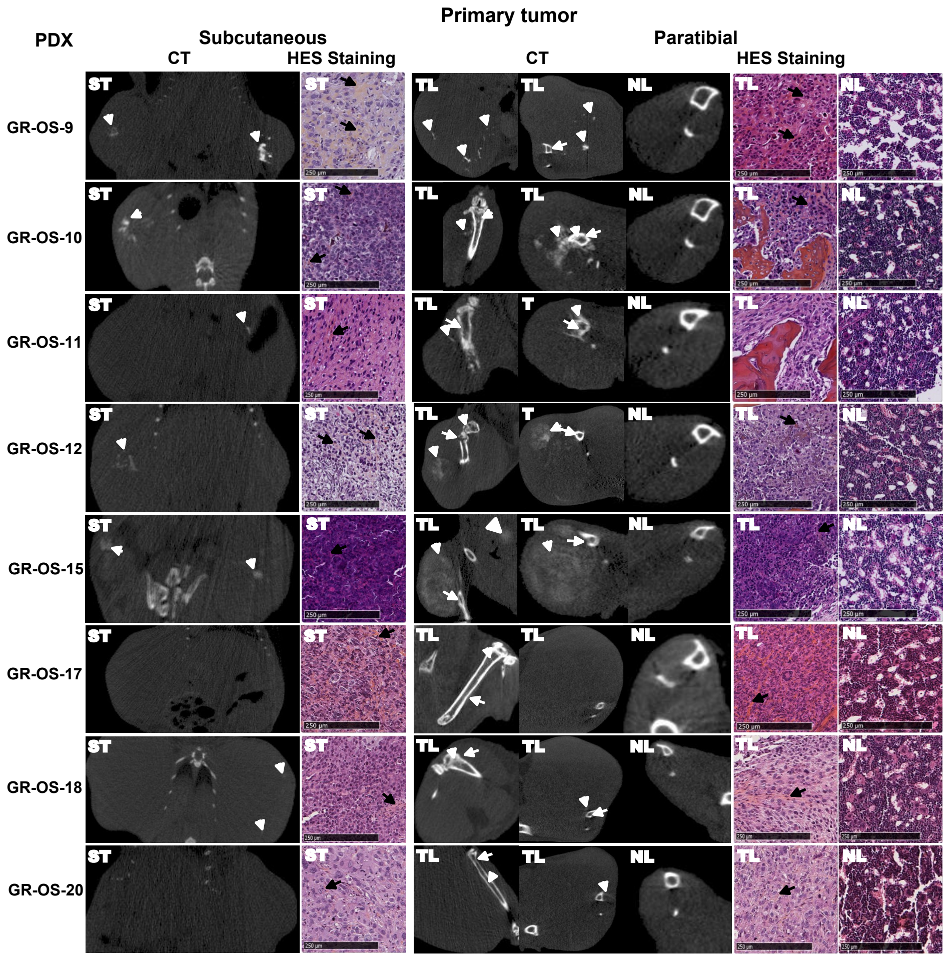

Supplementary Fig.S2: Radiological and morphological primary tumor PDX characteristics following subcutaneous and paratibial implantation. The short white

arrows show osteocondensation, the short/larger white arrows show metastasis osteocondensation, the long white arrows show osteolysis and the long black arrows show osteoid matrix (orange color). CT- CTscan; ST- Subcutaneous Tumor; TL- Tumor Leg; NL - Normal Leg.

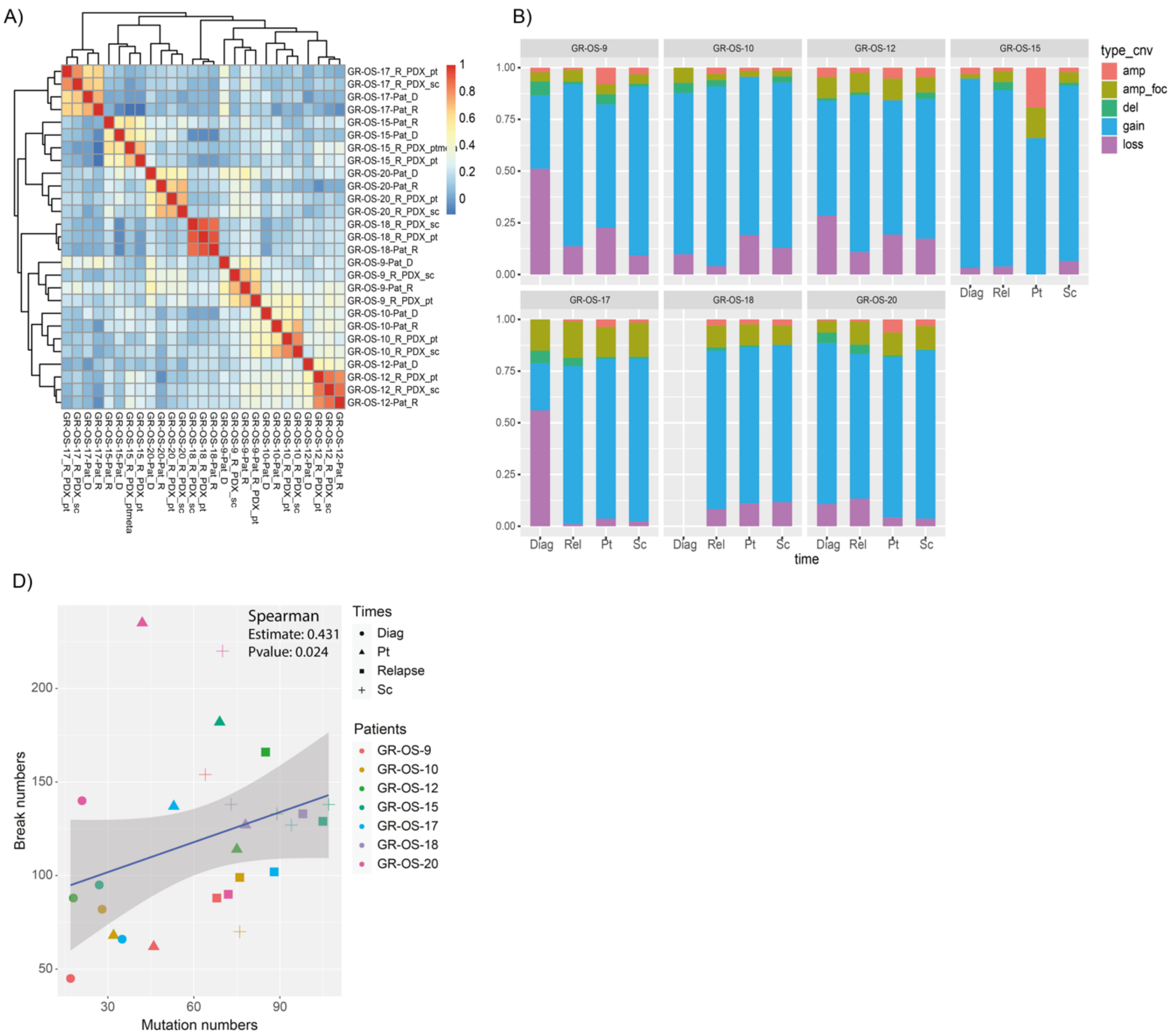

Supplementary Fig.S3: Copy number alterations through disease progression and in matched PDX models. A. Heatmap of the similarity of the genetic alterations (CNV and somatic mutations), it shows the proximity of the samples coming from the same patients through time and in the PDX. **B.** Barplot of the CNV type distribution through time by patient and in matched PDX models. **C.** Plot showing the significant linear correlation between the accumulation of CNV and of somatic mutations in all the samples. Diag- Diagnosis; Rel- Relapse; pt- paratibial; sc- subcutaneous.

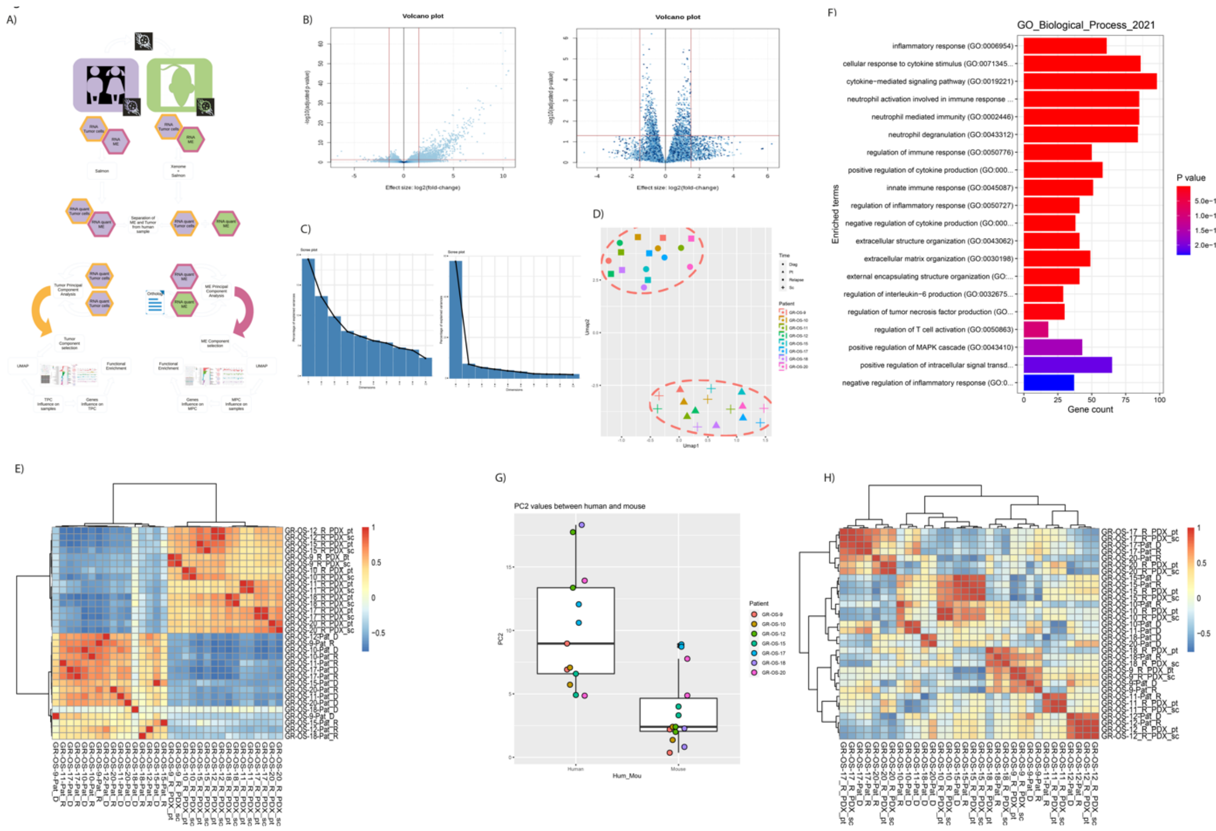

Supplementary Fig.S4: Identification of tumor cells components. A. Schematic view of the pipeline implemented to analyze separately the tumor and microenvironment transcriptomic program. **B.** Volcano plots before and after removing the genes differentially expressed between the human samples and the human fractions of the PDX samples**. C.** Screenplot of the variance explained by the principal components of the conserved genes (on the left) and of the removed genes (on the right). The first component of the removed genes represents the majority of the variance. **D.** The UMAP of the principal components before the removal of the genes differentially expressed. The removed genes being expressed only in the human samples, this result is expected. **E.** Correlation matrix for the expression of the removed genes show a specificity of expression for PDX and human samples. **F.** Functional enrichment analysis of the removed genes on the GO biological process database shows strong enrichment for TME genes **G.** Boxplot of the contribution to the second component of the human and PDX samples show a significant residual bias linked to the absence of TME in the PDX models. Therefore, the second principal component has been removed from the rest of the analysis. **H.** Correlation matrix of the gene expressions between all samples after the differential analysis and the removal of the differentially expressed genes specific to the patient TME. Diag- Diagnosis; Rel- Relapse; pt- paratibial; sc- subcutaneous.

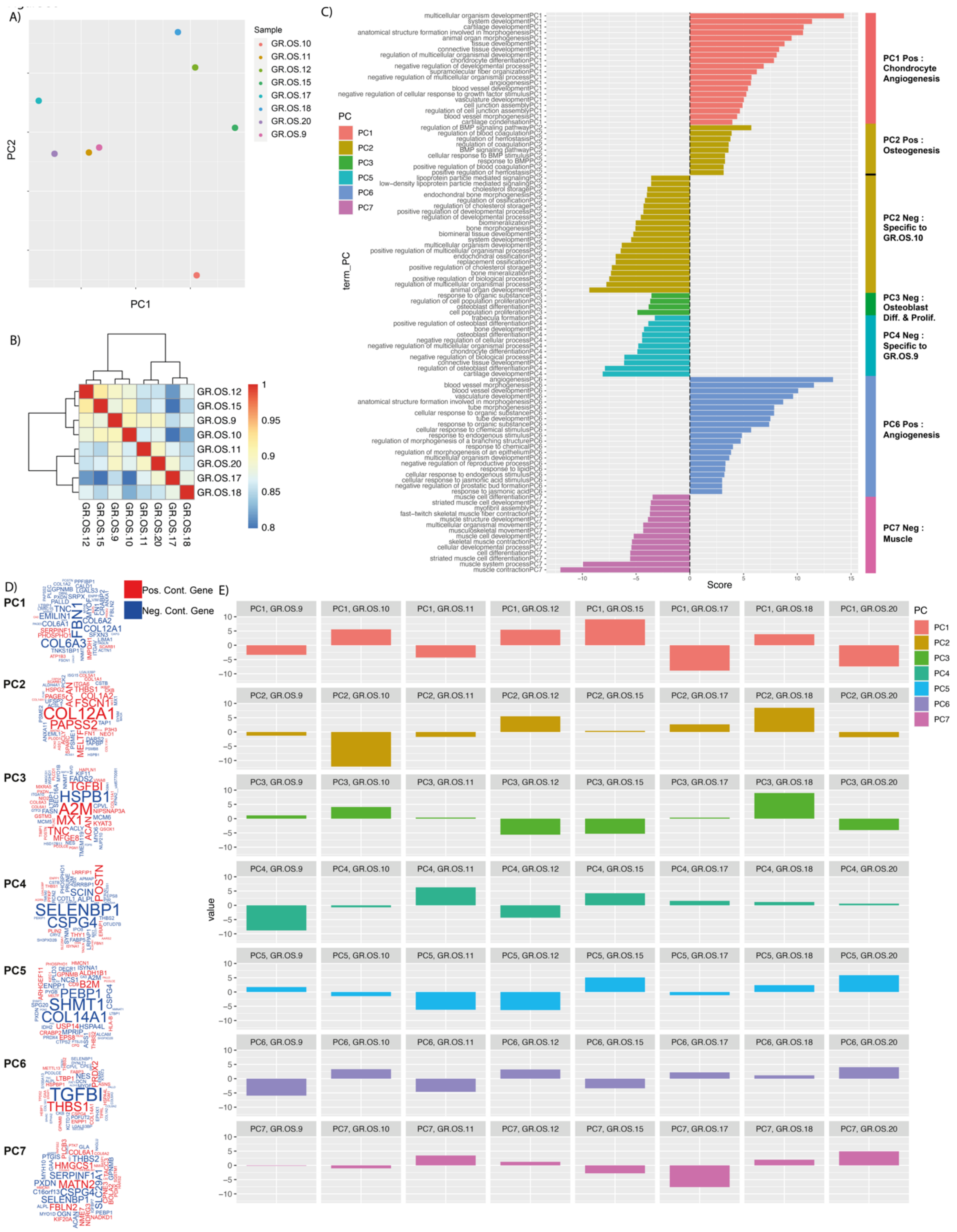

Supplementary Fig.S5: Identification of Proteomic components in Paratibial PDX models. A. Relative similarity of the 8 paratibial PDX models in the first 2 principal components of their proteomic quantification. **B.** Correlation matrix of the normalized protein intensities for the 8 paratibial PDX models. **C.** Word cloud illustrating the protein contributing the most to each component negatively (blue) or positively (red). **D.** Geneset enrichment analysis of each component based on gene contribution to proteomic based PC; Sign of the

enrichment score represents opposite functional enrichment describe by the same principal component.

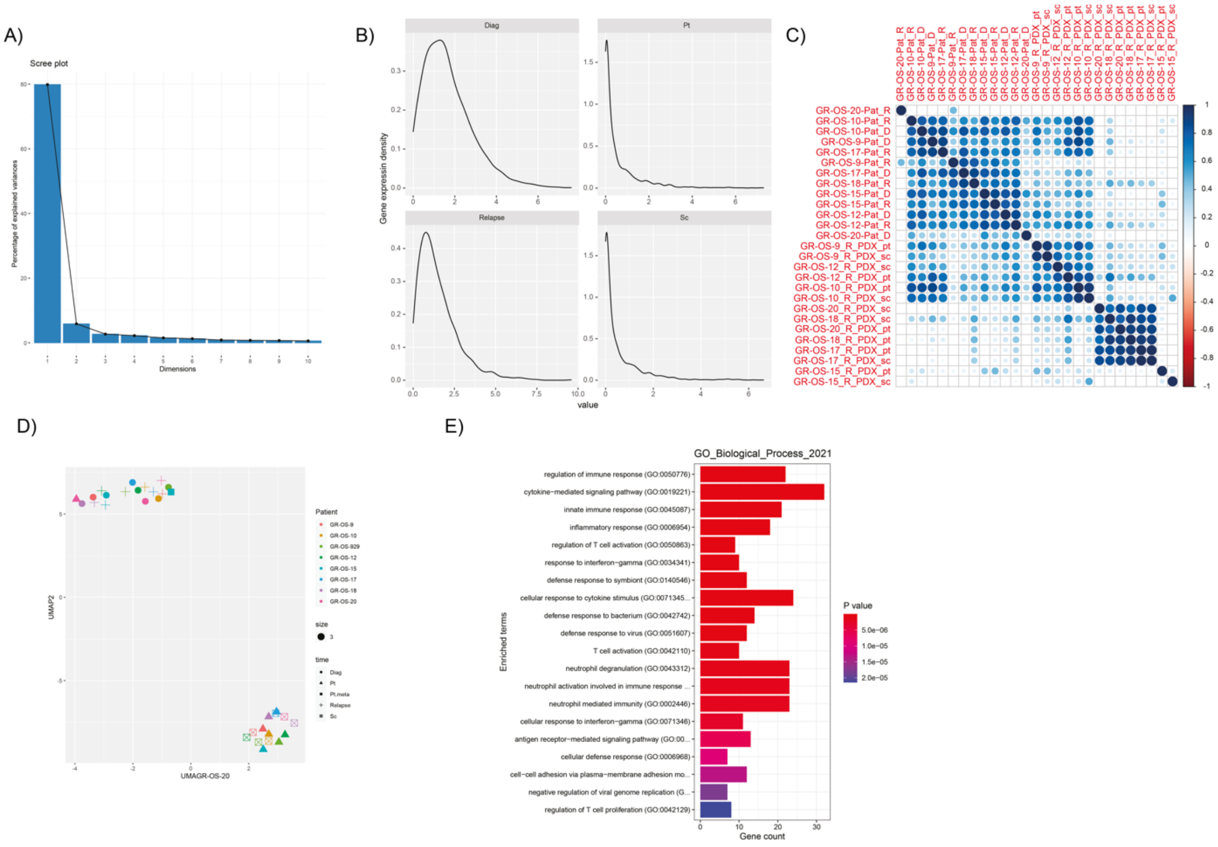

Supplementary Fig.S6: Identification of Microenvironment components. A. Barplot of the explained variance for the 10 first components of the removed genes. **B.** Density of the level of expression of the removed genes. **C.** Correlation matrix of the expression profiles of the removed genes, the human and PDX samples are separately clustered. **D.** UMAP of the principal components of the removed genes. **E.** Functional enrichment of the removed genes, most components are representing immunity expression. Diag- Diagnosis; Rel- Relapse; pt- paratibial; sc- subcutaneous; pt-meta- metastasis from paratibial PDX model.

Supplementary Table SI: Patients characteristics and PDX development. M=male, F=female, PHR=poor histological response (≥10% residual viable tumor cells), GHR=good histological response (<10% residual viable tumor cells), OS2006-first line treatment of osteosarcoma in France[2] which can combine different chemotherapies: M-EI: methotrexate-etoposide-ifosfamide, AP: doxorubicin-platinum, API-AI: doxorubicin-platinum-ifosfamide, EI: etoposide-ifosfamide, OS2TTP: second line treatment of relapsed osteosarcoma in France.

Randomizing HD-thiotepa:- high-dose-thiotepa, GEMOX:- gemcitabine-oxaliplatine, VP16-Carbo:- etoposide-carboplatin, EDX:- cyclophosphamide, CR: complete response, PR: partial response, SD: stable disease, PD: progressive disease, UKN: disease not yet evaluated under therapy, PD: progressive disease, R: relapse, Met: metastatic, Px- *in vivo* passage n°x.

Patient	GR-OS-9	GR-OS-10	GR-OS-11	GR-OS-12	GR-OS-15	GR-OS-17	GR-OS-18	GR-OS-20
**at diagnosis**								
Age (years)	14	16	13	16	16	11	14	16
Gender	M	M	F	F	M	M	M	M
Primary tumor localisation	femur	femur	femur	femur/tibia	femur	humerus	femur	femur
Histology	osteoblastic	telangiectatic	fibroblastic	osteogenic osteosarcoma	chondroblastique	giant cell osteosarcoma	osteoblastic	osteoblastic
Metastatic status	no	lung	no	osteosarcomatosis: bone, lung, lymph nodes, muscles, sub-cutanéous	osteosarcomatosis: bone, lung	no	lung	bone, lung
First line chemotherapy	MEI/AP	M-EI	MEI/AP	API-AI	MEI	MEI	MA	MEI/AP
Chemotherapy after early progression	na	API-AI/EI	na	na	API-AI/AI-AP-EI	na	AP/EI	
Surgery of primary tumor	R0	R0	R1/tumor micro-embolism	no surgery	R0	R0	R1/tumor micro-embolism	R0
Histological response	Poor	Good	Poor (28% viable cells)	NA	Good 0%	Good 0%	Poor (50% viable cells)	Poor (16% viable cells)
nb of Progression/relpase before MAPPYACTS inclusion	1 (lung at M18)	1 early local progression at W7	1 (lung at M14)	0	1 early local progression at W7	3 (lung M17; lung M36; bone M47)	2 (loc/met PD at M3 ; Lung Met at M11)	
Treatment for relapse before MAPPYACTS	EI - surgery	surgery	M14 EI	0	0	M17 AP-HD thiotepa M36 surgery M47 surgery oral Cy-rapamune		
**Cumulative chemotherapy dose before MAPPYACTS sample**								
Methotrexate (g/m²)	96	36	84	0	36	228	56	84
Doxorubicine (mg/m2)	350	240	300	360	420	280	280	350
Cisplatinum (mg/m2)	600	300	600	400	360	480	360	600
Ifosfamide (g/m2)	72	84	48	60	60	60	48	24
Etoposide (mg/m2)	1600	1800	1200	0	600	1200	1200	600
**At the relapse included in MAPPYACTS**								
Age (years)	16	17	13	16	17	19	14	20
nb of the relapse/progression	1 after chemo	2	2	1	2	4	2	1
Type	metastatic	local/metastatic	metastatic	local/metastatic	metastatic	metastatic	local/metastatic	metastatic
Localisation	lung	liver, lung, bone	lung, lymph node	all sites	bone, lung	lung/heart	nodes, lung	isolated lung nodule
**Time from initial diagnosis (Months)**	**M30**	**M11**	**M17**	**M5**	**M11**	**M111**	**M16**	**M55**
Sample	lung nodule resection after EI	liver nodule biopsy	lymph node biopsy	lymph node biopsy	lymph node biopsy	lung nodule resection	intra-muscular nodule	lung nodule complete resection
**Relpase treatment after MAPPYACTS**	high-dose thiotepa	AP	Lenvatinib	EI	gemcitanib/sorafenib	pazopanib	cabozantinib	0
**Outcome**								
Relapse/progression	M34	M15	M20	constant PD	constant PD	M122	M20	0
Furthers treatments	Lenvatinib	pazopanib	pazopanib	0	0	temozolomide	oral VP16	0
**death**	**M49**	**M24**	**M27**	**M9**	**M12**	**M124**	**M21**	**Alive in RC2 at M81**

Supplementary Table SII: Osteosarcoma PDX Tumor characteristics for different passages and at sacrifice day for subcutaneous and paratibial models.

PDX ID	Implantation site	First tumor detection median time (days)	Median time Detection-Sacrifice (days)	TD (doubling time - days) P0	TD (doubling time - days) P2	% Tumor take	Tumor aspect	Growth localization	PDX Cells culture
**GR-OS-9**	Subcutaneous	24 (11-67)	28 (10-48)	5.2	4	100	Calcified (++)	in the two flanks	Growth stopped P2<br>+++ cells adherence
	Paratibial	27 (11-42)	26 (13-28)	-	-	56	Calcified (+++)	Tibia, Femur and pelvis (close to the bone)	Growth stopped P1<br>+++ cells adherence
**GR-OS-10**	Subcutaneous	25 (19-26)	35 (22-52)	-	5.5	100	Calcified (++)	in the two flanks	Growth after P2<br>++ cells adherence
	Paratibial	69 (40-97)	58 (34-80)	-	-	100	Calcified (++++) and viscous	Tibia (Inside the bone)	Growth after P2<br>++ cells adherence
**GR-OS-11**	Subcutaneous	25 (24-109)	29 (21-42)	14	7	100	Calcified (+)	Tibia (Inside the bone)	Growth after P2<br>+ cells adherence
	Paratibial	25 (19-42)	27 (21-62)	-	-	60	Calcified (++)	Tibia (Inside the bone)	Growth after P2<br>+ cells adherence
**GR-OS-12**	Subcutaneous	14 (13-52)	19 (14-29)	8	5	100	Calcified (++)	in the two flanks	Growth after P2<br>++ cells adherence
	Paratibial	33 (11-42)	20 (17-22)	-	-	60	Soft exterior and calcified (+++)	Tibia and femur (Inside the bone)	Growth after P2<br>++ cells adherence
**GR-OS-15**	Subcutaneous	15 (10-27)	15.5 (7-28)	5	4.2	100	Calcified (+++)	in the two flanks	Growth after P2<br>++ cells adherence
	Paratibial	27(19-44)	28 (18-41)	-	-	83	Calcified (+++++)	Tibia (Inside the bone)	Growth after P2<br>++ cells adherence
**GR-OS-17**	Subcutaneous	42(35-70)	94(12-125)	9	6.5	100	No Calcified	in the two flanks	Slow Growth (P0)
	Paratibial	74,5(70-93)	71(12-95)	-	-	80	Calcified (+)	Tibia (Inside the bone)	Slow Growth (P0)
**GR-OS-18**	Subcutaneous	19,5(18-21)	9(8-10)	-	5	100	Calcified (++)	in the two flanks	Growth after P2<br>+ cells adherence
	Paratibial	19(19-22)	32(20-48)	-	-	100	Calcified (++)	Tibia and Femur (Inside the bone)	Growth after P2<br>+ cells adherence
**GR-OS-20**	Subcutaneous	93,5(27-156)	23(17-63)	22	11	100	Calcified (+)	in the two flanks	-
	Paratibial	135(53-217)	52(43-60)	-	-	50	Calcified (+)	Tibia (Inside the bone)	-

Supplementary Table SIII: Removed Genes from inferred ME to study Tumor cells expression.

Removed Genes from inferred ME to study Tumor cells expression

C1QA

C1QB

C1QC

SFTPC

TYROBP

RNASE1

MMP9

CD74

MS4A6A

HLA-DRB5

VWF

HBA1

FCGR3A

CCL21

RNASE6

PLVAP

CD14

S100A9

MS4A7

VSIG4

CD53

HBA2

GIMAP4

TREM2

MS4A4A

ECSCR

CXCL10

MPEG1

SLCO2B1

GSTM1

GIMAP7

GIMAP5

LY86

TMEM176B

TCIM

CD163

CLDN5

LYZ

CCR1

LILRB4

RPL10L

HCST

FCGR2B

CLEC14A

CD300A

CD93

FPR3

GGT5

FCGR1A

S100A8

CD34

PRND

MNDA

CDH5

CD33

NKG7

CD84

LY6H

MYCT1

TPSAB1

COX4I2

CCL8

SCARF1

RUFY4

IL10RA

LGALS9

SAMSN1

TM4SF18

IGSF21

SIGLEC1

JAML

CD68

TPSB2

ITGAX

LILRB2

GJA4

GIMAP8

SOX7

FGL2

HLA-DQA1

CSF3R

GRAP

DLL4

GNA15

OSCAR

IL18

APOBR

ACP5

CD3D

SLAMF8

APLNR

CD2

CD3E

IL1RN

ST14

ADA2

KCNQ1

PLA2G7

LYVE1

C1orf162

CXCR4

CD209

CLEC7A

SLAMF7

IL2RG

RASAL3

KCNK17

APOC1

JCHAIN

LILRB5

CD48

ARHGAP9

FCGR2A

MMP1

C7

CLEC4A

KDR

PCDH12

DIPK2B

NPR1

SLC37A2

ESAM

TREM1

CD200

EGFL7

SOX18

FGD2

MMRN2

FOS

LRRC25

CHST1

FOLR2

TM4SF19

CD8A

TMEM273

CD4

SCGB3A1

SIGLEC9

SRGN

CXCL9

SHE

TNFRSF1B

CIITA

C15orf48

ADAMDEC1

SDS

SEMA6B

SIGLEC7

PSTPIP1

ODF3B

CD300C

APOC2

MALL

LILRB3

SERPINA1

HBG2

GZMH

MMP19

PTPRCAP

C3

MS4A14

IGLL5

EBI3

GIMAP6

PRF1

HP

GIPC3

CLEC10A

VSIR

APOE

CLEC3B

TFEC

TNFSF13B

GIMAP1

BCL2A1

KANK3

IGSF6

BCL6B

MSR1

SLC49A3

CCL24

FAM107A

IFI30

NAPSA

EGFL6

F2RL2

ROBO4

ARHGAP4

ADGRL4

CNMD

FABP4

RUBCNL

VAMP8

ICAM1

ADGRF5

TESC

HIGD1B

CCRL2

FCER1A

DCSTAMP

RASGRP4

CD180

SOX17

NRROS

TSTD1

FLT4

LAIR1

MYL1

SUCNR1

APOBEC3A

SHANK3

EDN1

GNLY

LRRC36

FLT1

SELPLG

SIRPB2

SUSD2

MMRN1

LILRA5

TINAGL1

RIMS1

CCL13

SNX20

N4BP3

ZNF467

CCND2

LCK

TMEM88

STAB1

DNASE1L3

CDH6

CD300LF

DEPP1

FAM167B

CSF2RB

SFMBT2

EVI2B

CLEC4E

NCF4

TNFSF8

FPR1

SIGLEC14

NFAM1

ADIRF

MARCO

ALOX5

PYHIN1

LAPTM5

PDGFB

TMEM176A

GALNT18

COL18A1

CD69

RAB20

BTNL9

CD7

ADGRE2

CALCRL

CFD

MS4A15

CD1C

CETP

CD22

POTEJ

TMEM255B

TYMP

RIPK3

PLPP2

ATP6V0D2

MATK

MRO

LMO2

TRPC6

PTGS1

TNFRSF10C

TMEM233

ADAM8

MUSTN1

LHX8

HSD3B7

CPA3

FCN3

ITGB2

TPPP3

MLPH

ALDH1A1

PPP1R16B

CREG2

MMP15

ADGRG1

NLRC4

PCDH1

LAMC3

HSD17B14

LILRA6

CCM2L

DPEP2

SCN4B

RASGRP3

DNAAF1

TNFRSF11A

GSDMA

GRAPL

NLRP3

APOD

TFF3

CD28

DOK2

SLCO2A1

PDK4

ITIH5

HSD11B1

MANSC1

TLR7

AVPR1A

FERMT3

P2RY13

MPZL2

CD27

IL10

MEIKIN

F5

SH3TC1

SPARCL1

APBB1IP

ARL11

HAMP

IL4I1

IL12RB1

RAPGEF5

IL1B

FAM124B

DNAJC5B

C16orf89

MMP7

NCF1

PAEP

ABCG1

RAMP2

CFI

IL2RA

PILRA

CLECL1

TNFSF12-TNFSF13

ENTPD1

RBP7

EXOC3L1

INHBB

SNCG

PINLYP

ESM1

IL33

VMO1

TAL1

GZMB

GRK5

SLAMF6

DENND1C

TNFSF13

SCIMP

PARM1

ANGPT2

IGF2

PEAR1

SERPINB9

WSCD1

VENTX

CLIC2

SOCS3

CRHBP

TNFRSF4

NRARP

CFP

SLC22A1

LTF

ZFP36

VSIG2

H3-5

PPP1R14A

GIMAP2

MYZAP

GRAP2

BIRC3

LDLRAD2

RASSF4

CEACAM21

CYBB

JAK3

SIRPG

TBX1

INMT

CARD11

COL8A1

BLNK

LRRC70

CPLX1

SLC2A9

GMFG

CASP10

LGI4

CDH1

FCER2

FCHO1

RND1

CSF1R

DAPP1

PKD2L1

GPR34

PTPN22

SPN

SLC15A3

FOLH1

CD300E

MAP1LC3C

HYAL1

PYCARD

LAT2

CYTH4

PTGIR

GABRD

TCIRG1

HSPA6

MEFV

HAVCR2

SECTM1

RGCC

ADAM28

STAP2

KLRB1

SORL1

SLC11A1

SIGLEC10

ARHGEF15

EFCC1

PRR29

COL5A3

PLEK

SASH3

RNASE2

GPR65

REM1

PCLO

WFDC2

CD248

C5AR1

CASS4

CLDN7

SPOCK2

DMKN

SIRPA

BIN2

CDCP1

ABI3

GIPR

CLEC5A

KIT

RASIP1

OLR1

COL4A2

SYTL1

NPDC1

PAMR1

MEGF6

NOVA2

GAP43

PLCB2

SULT1C4

WDFY4

ADAP2

LY9

IL2RB

TLE2

DOCK6

CD3G

HSPA12B

LAMP3

STEAP4

PTPRE

ACVRL1

GPR84

PXDN

CASP1

VNN1

PLA2G4C

FYB1

KIAA1755

HBB

PRAM1

USHBP1

FCER1G

PRSS36

IL18R1

FBP1

DUSP4

CASP5

CD247

SIT1

CLDN23

CRACR2B

RBM47

COL4A1

MYH11

ZAP70

CYGB

TRPM2

SLA

RGS10

PDE1B

CD79B

GZMK

PKIB

PHYHD1

MYO1G

PTPRC

SH2D3C

SIGLEC15

CYP2S1

CD37

TBXA2R

HS3ST2

SPRY4

CD200R1

HCLS1

AOAH

EPAS1

ADAMTSL2

MOXD1

PTAFR

PIK3AP1

ADORA2A

IGSF22

ITGA8

ADAMTS14

S1PR1

FBXO48

ANPEP

HTRA3

KMO

LCP2

MMP11

IL32

KCNE3

CAPN3

CRYBB1

FOXP2

DISC1

NUAK2

S100B

HEYL

RPS6KA2

FAM110D

SERPINF2

DTX1

CYTL1

ARHGAP30

ZNF366

ENG

RNF144B

RASD1

HCK

ZC3H12D

TRPV2

TMEM86A

A4GALT

RCSD1

C1QTNF3

HSD11B2

TLR1

CD40

SCNN1A

FGD5

PTPN6

ELN

ZNF154

LPAR6

ST6GAL1

TNFSF10

ICAM2

PRDM1

LPAR5

CDA

DUSP5

SLC2A3

INHBE

SERPING1

RGS18

RASGRF2

AATK

NCKAP1L

FGR

TLCD2

LAMA5

KCNK6

RNASET2

STC1

EGR1

NME1-NME2

CTSK

IL18BP

ITGAM

FZD4

RAC2

CST3

TNFRSF14

GBGT1

RASL11B

CYBA

SLPI

SULF2

THEMIS2

BICDL1

PLXDC1

EPS8L1

TNFAIP8L2

GAL3ST4

GBP4

FILIP1

BTG2

SLC9B2

SPINT2

RSC1A1

FBLIM1

PGF

KCNJ5

PRRG2

CAMK4

EEPD1

SIPA1

OASL

ACE

EPB41L4A

ITGB3

HMOX1

PDZD4

CRYGS

PLTP

HIC1

COL8A2

FOSB

HPGDS

P2RY6

SLC22A18AS

TIE1

HAPLN1

LYPD5

SULT1B1

GALNT6

MAP3K8

CTSS

JUNB

SPI1

C19orf38

RASL10A

ADCY4

ARHGDIB

MECOM

ARAP3

TNFRSF21

PTPN7

TLR2

AMDHD1

PREX2

HOPX

PCNX2

LXN

PLXND1

NOTCH1

P2RY10

AMIGO2

PLAAT4

APLN

IKZF1

APOBEC3D

FAM78A

MX2

SEMA3F

CCR5

SUGCT

MAP4K1

NCF2

ABCC9

FRAT1

NTRK2

JCAD

SCGB3A2

CDC42EP2

DPEP1

FXYD2

FAM78B

OLFML2A

MPZ

MFNG

ITIH4

PSD4

TAGAP

ALOX5AP

CCL2

SLCO4A1

PLB1

NPNT

TF

TNFAIP3

SEPTIN1

KLHL6

TMEM150B

PLD4

CISH

PARP15

FCMR

BCAM

UNC13D

KIF17

ASAH2

PTPRB

MAPK11

BTK

TNFAIP8L1

NRXN2

PRCD

NAALADL1

SAT1

RBPMS

CXCL16

FCGRT

CPM

SOX13

SHROOM4

OAS1

SIPA1L2

PTP4A3

NPL

PPARG

TBC1D10C

SGIP1

AGT

RHPN1

TMC8

VAV1

TRIM22

TLCD4

KIAA0040

CTSB

RILP

C1S

MLKL

GDF15

SAMHD1

HYAL2

TRAF3IP3

GPR160

DYSF

AKNA

FRZB

HHEX

CRISPLD2

CEBPA

BEST1

TRIM34

LYL1

TOR4A

GPR4

ITGAL

PTPRO

POLN

ZSCAN22

FAM20A

FJX1

SCN1B

ARHGEF28

F11R

EVA1C

RASA4B

ZNF799

CLDN11

LY96

SH2B3

TNS1

ITPKB

HK3

TPP1

C5AR2

PLXNA2

AFAP1L1

JUP

A2M

KCNJ8

CSTA

P2RY1

IFITM1

THBS1

PLLP

ZNF347

NFIX

S100A3

TMC4

SHFL

DOCK9

TNFRSF12A

ZNF554

SOCS2

C12orf75

CAP2

IRS2

RHOF

DIPK1B

IGFLR1

RNF152

ISG20

INAFM1

TMEM37

CA2

ECE1

CCDC88B

IGFBP7

NBEAL2

CYP1B1

PLEKHG2

PDE9A

VWA5A

ADM

PIK3IP1

PALMD

MVP

RNF125

RESF1

PLXNB3

ATP6V1B2

NID1

CPT1C

MCAM

NFATC2

IL17RA

PIK3R5

RENBP

DNAH1

PALM

SPRY1

CCDC102B

VASH1

TTYH2

FKBP5

TEK

OTULINL

CALHM6

DUSP1

HLX

TCN2

EDNRA

INSR

UBE2S

NHSL2

CRISPLD1

ANK2

ZFP36L2

EOGT

RAB11FIP4

INKA2

LAMB3

SIGIRR

NGFR

JAG1

IFIT1

ZNF334

SLC16A5

GSDMB

ARRDC2

PTPRJ

ETS1

ITGA2

NID2

DBP

STOM

CACNA2D4

MBP

LDB2

SLC22A18

ITPRIP

TNS2

CD72

CEMIP

GNRH1

IRF7

PLEKHO2

ZBTB46

LAT

ADAM32

RASSF2

FLT3LG

B3GNT5

GAB3

LCP1

NFKBIA

ITGA1

PGM2L1

TGM2

APOL4

TBC1D30

NBL1

ZGLP1

TSPAN2

NDUFA4

OBSCN

FTL

CXCR5

METTL7A

CNTNAP1

CDH11

CDH15

HEY1

PPP1R15A

ADAMTS7

ZNF385A

SEMA4A

CEACAM19

SLC9A3R2

NOTCH3

ATG16L2

PRKCH

MDFI

PPP1R32
